# Estimation of the diaphragm neuromuscular efficiency index in mechanically ventilated critically ill patients

**DOI:** 10.1186/s13054-018-2172-0

**Published:** 2018-09-27

**Authors:** Diana Jansen, Annemijn H. Jonkman, Lisanne Roesthuis, Suvarna Gadgil, Johannes G. van der Hoeven, Gert-Jan J. Scheffer, Armand Girbes, Jonne Doorduin, Christer S. Sinderby, Leo M. A. Heunks

**Affiliations:** 1 Department of Anesthesiology, Radboud University Medical Center, Nijmegen, The Netherlands; 2 Department of Intensive Care Medicine, Amsterdam UMC, Vrije Universiteit Amsterdam, Postbox 7057, 1007 MB Amsterdam, The Netherlands; 3 Department of Intensive Care Medicine, Radboud University Medical Center, Nijmegen, The Netherlands; 4 Department of Anesthesiology, University Medical Center Utrecht, Utrecht, The Netherlands; 5 Department of Neurology, Donders Institute, Radboud University Medical Center, Nijmegen, The Netherlands; 6 Department of Critical Care Medicine, St. Michael’s Hospital, University of Toronto, Toronto, ON Canada

**Keywords:** Diaphragm dysfunction, Neuromuscular efficiency index, Mechanical ventilation, Partially supported mode, Diaphragm electromyography, Monitoring

## Abstract

**Background:**

Diaphragm dysfunction develops frequently in ventilated intensive care unit (ICU) patients. Both disuse atrophy (ventilator over-assist) and high respiratory muscle effort (ventilator under-assist) seem to be involved. A strong rationale exists to monitor diaphragm effort and titrate support to maintain respiratory muscle activity within physiological limits. Diaphragm electromyography is used to quantify breathing effort and has been correlated with transdiaphragmatic pressure and esophageal pressure. The neuromuscular efficiency index (NME) can be used to estimate inspiratory effort, however its repeatability has not been investigated yet. Our goal is to evaluate NME repeatability during an end-expiratory occlusion (NMEoccl) and its use to estimate the pressure generated by the inspiratory muscles (Pmus).

**Methods:**

This is a prospective cohort study, performed in a medical-surgical ICU. A total of 31 adult patients were included, all ventilated in neurally adjusted ventilator assist (NAVA) mode with an electrical activity of the diaphragm (EAdi) catheter in situ. At four time points within 72 h five repeated end-expiratory occlusion maneuvers were performed. NMEoccl was calculated by delta airway pressure (ΔPaw)/ΔEAdi and was used to estimate Pmus. The repeatability coefficient (RC) was calculated to investigate the NMEoccl variability.

**Results:**

A total number of 459 maneuvers were obtained. At time *T* = 0 mean NMEoccl was 1.22 ± 0.86 cmH_2_O/μV with a RC of 82.6%. This implies that when NMEoccl is 1.22 cmH_2_O/μV, it is expected with a probability of 95% that the subsequent measured NMEoccl will be between 2.22 and 0.22 cmH2O/μV. Additional EAdi waveform analysis to correct for non-physiological appearing waveforms, did not improve NMEoccl variability. Selecting three out of five occlusions with the lowest variability reduced the RC to 29.8%.

**Conclusions:**

Repeated measurements of NMEoccl exhibit high variability, limiting the ability of a single NMEoccl maneuver to estimate neuromuscular efficiency and therefore the pressure generated by the inspiratory muscles based on EAdi.

**Electronic supplementary material:**

The online version of this article (10.1186/s13054-018-2172-0) contains supplementary material, which is available to authorized users.

## Background

Diaphragm dysfunction frequently develops in mechanically ventilated intensive care unit (ICU) patients and is associated with adverse clinical outcomes including prolonged mechanical ventilation and mortality [1–7]. It appears that non-physiological diaphragm activity plays an important role [8], in which both disuse atrophy resulting from ventilator over-assist [3, 9, 10] and high respiratory muscle effort resulting from ventilator under-assist [11–13] have been associated with diaphragm dysfunction in ICU patients. Therefore, there is a strong physiological rationale for monitoring diaphragm effort [14–16] and titrating support to maintain respiratory muscle activity within physiological limits [17].

Variations in esophageal pressure (Pes) during breathing have been used for decades to quantify breathing effort. Recently, two state-of-the-art papers reviewed the technical and clinical aspects of esophageal pressure (Pes) monitoring in ICU patients [18, 19]. Limitations of this technique include strict control of balloon inflation volume and complexity of signal interpretation, in particular when expiratory muscles are recruited.

Diaphragm electromyography (EMG) is an alternative technique used to quantify breathing effort in ICU patients [15]. Strong correlation has been reported between the electrical activity of the diaphragm (EAdi) and transdiaphragmatic pressure (Pdi) or Pes [20, 21]. The neuromechanical efficiency (NMEoccl), defined by delta airway pressure (∆Paw) divided by ∆EAdi measured during an end-expiratory occlusion, has been used to estimate the inspiratory effort breath by breath [21, 22]. This ratio describes how much pressure can be generated for each microvolt of EAdi signal, in other words how efficient the diaphragm is in generating pressure for a certain amount of electrical activity. This is of potential interest for monitoring diaphragm function (over time) and helps to titrate ventilatory support in order to minimize diaphragm dysfunction resulting from ventilator over-assist and under-assist [17]. Today, this index has only been evaluated in studies including limited numbers of patients [21–24] and the repeatability, an essential characteristic for a diagnostic tool, has not been investigated at all. Therefore, the aim of our study was to evaluate the NMEoccl repeatability in mechanically ventilated ICU patients and its use to estimate the maximum inspiratory pressure generated by the inspiratory muscles (Pmus).

## Methods

### Study design and population

This prospective cohort study was performed in an academic ICU. Adult patients, with a dedicated EAdi catheter (Maquet critical care, Solna Sweden) in situ and mechanically ventilated in neurally adjusted ventilator assist (NAVA) mode were eligible for inclusion. The institutional ethical committee approved the study protocol and informed consent was waived due to the non-invasive nature of the study and negligible risks.

### Study protocol

The EAdi catheter was positioned according to the manufacturer’s instructions using a dedicated software tool on the Servo-i ventilator. The catheter position was verified before data acquisition. When patients exhibited a stable breathing pattern (i.e. no coughing, hiccups or disproportional differences in respiratory rate), measurements to assess respiratory muscle function were performed: (1) an end-expiratory occlusion maneuver (obtained by activating the expiratory hold button on the ventilator for one inspiratory effort) for measurement of NMEoccl and (2) a “zero assist breath” in which inspiratory support was decreased to zero for one single breath to calculate patient-ventilator breath contribution (PVBC) [25, 26]. Both maneuvers were repeated five times with at least a one-minute interval. The number of breaths between maneuvers was variable to prevent anticipation by the patient. Measurements were recorded and stored for offline analysis at time *T* = 0 and after 12 h (*T* = 12), 24 h (*T* = 24) and 72 h (*T* = 72).

### Data acquisition

EAdi, flow and Paw waveforms were acquired from the Servo-i ventilator via a RS232 serial port connected to a laptop with dedicated software (Servo Tracker version 4.1, Maquet, Solna, Sweden). Maximum inspiratory pressure (MIP) measurements were performed with a manovacumeter (Micro Respiratory Pressure Meter, Carefusion, Yorba Linda, CA, USA) connected to the endotracheal tube [27].

### Data analysis

A software routine developed for MatLab (version R2016b, MathWorks Inc., Natick, MA, USA) was used for offline analysis.

**Fig. 1:**
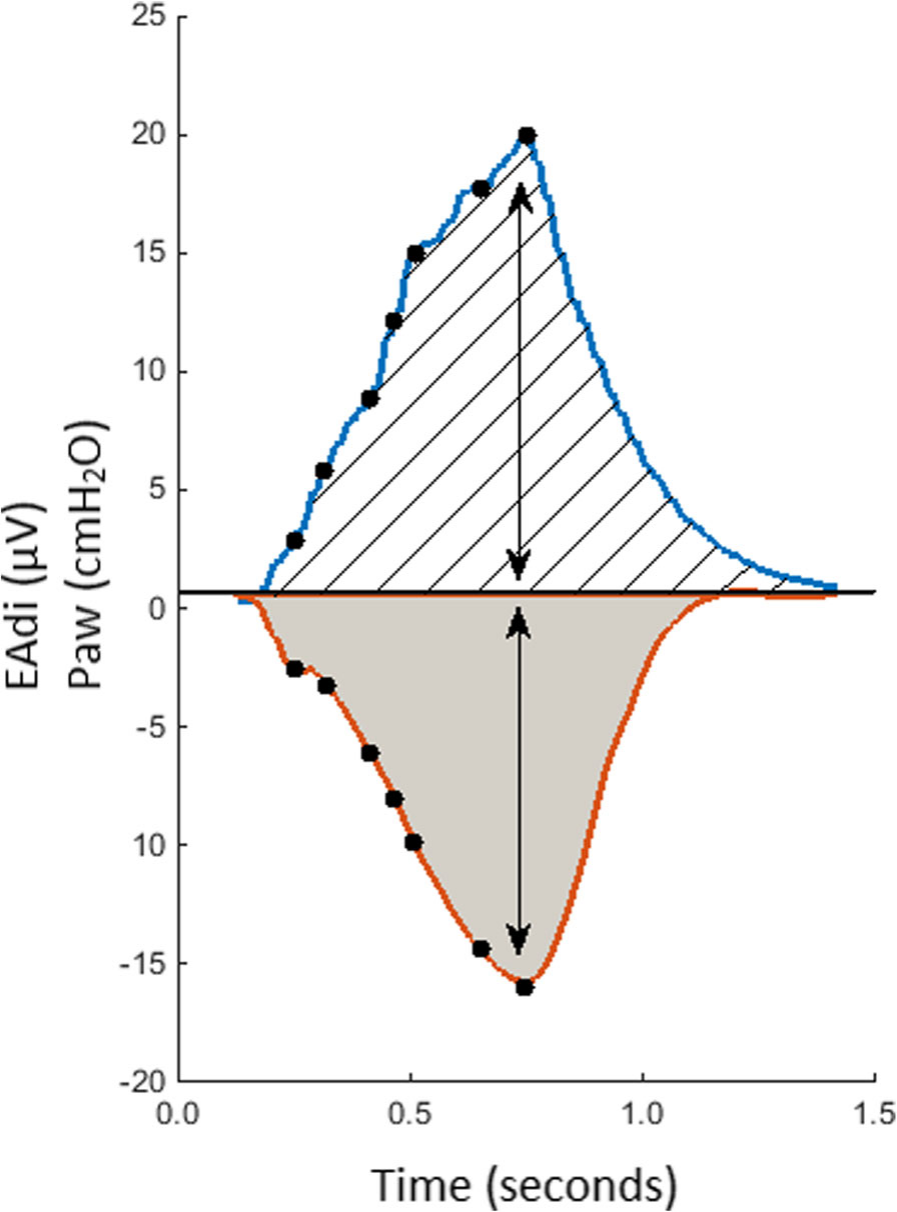
Example of a single neuromechanical efficiency index during an end-expiratory occlusion (NMEoccl) maneuver. The blue line represents the electrical activity of the diaphragm (EAdi) signal expressed in microvolts. The orange line represents the airway pressure (Paw) expressed in centimeters of water. As described above, the NMEoccl was calculated in three different ways, with the calculation based on (1) delta peak values of electrical activity of the diaphragm (EAdi) and Paw, shown as arrows; (2) area under the curve (AUC) of the EAdi and Paw signal, shown by diagonal lines and gray area, respectively; (3) using fixed points (steps of 3 μV) on the EAdi curve (during inspiration) and corresponding Paw, shown as black dots

NMEoccl was calculated in three different ways (Fig. 1): (1) by dividing ∆Paw by ∆EAdi [21], in which ∆Paw is the difference in pressure between the lowest Paw during end-expiratory occlusion and the preceding end-expiratory pressure level, (2) by dividing the area under the curve (AUC) of Paw and EAdi, and (3) by dividing Paw and EAdi at fixed points (steps of 3 μV) on the EAdi waveform. The inspiratory time (Ti) was defined as the period between the onset of EAdi and 70% peak of EAdi. To investigate the effect of an occlusion on the inspiratory time, we compared Ti during occlusion with the Ti of three preceding unloaded breaths. To estimate Pmus under clinical conditions, the mean EAdi of five tidal breaths (before the end-expiratory occlusion) was multiplied by NMEoccl/1.5 [21]. The correction of 1.5 compensates for the fact that in the presence of flow, the diaphragm generates less pressure for the same EAdi than during an occlusion [21]. The tension-time index (TTI) was calculated as Pmus/MIP multiplied by the ratio of Ti to total respiratory cycle time (T_tot_) [28].

### Statistical analysis

Statistical analysis was performed with GraphPad PRISM (version 5.03 for Mac/Windows, Software Inc. San Diego, CA, USA). Data were analyzed as median ± interquartile range (IQR), except as stated otherwise. Statistical significance was indicated by a *p* value <0.05.

The repeatability coefficient (RC) represents the absolute value by which two repeated measurements in one subject will differ in 95% of cases. The formula developed by Bland and Altman was used to calculated RC:

1.96 × √2 × Within-subject standard deviation (SD) [29].

One-way analysis of variance (ANOVA) was used to obtain the within-subject SD with the subject as dependent factor and the repeated NMEoccl measurements as independent factors. Since the NMEoccl variability increased as the magnitude of NMEoccl increased, the ratio of a single NMEoccl measurement to the mean NMEoccl of five repeated measurements was used (see Additional file 1) [29]. The correlation coefficient with repeated observation was used to investigate the within-subject correlation between Paw and EAdi [30, 31] (IBM SPSS Statistics version 22).

In addition, to obtain the within-subject NMEoccl variation, per patient for each time point a coefficient of variation (CoV) was calculated by the ratio of the within-subject SD to the mean. The median CoV was used to divide the study population into two groups with CoV higher or lower than median. The paired *t* test was used to test for differences in clinical parameters (that might affect NMEoccl variability) between both groups.

The Pearson correlation coefficient was calculated to test correlation between Ti and NMEoccl, and between TTI and Pmus. One-way ANOVA was used to investigate the changes in NMEoccl and inspiratory muscle pressure over time.

## Results

Table 1 shows the main characteristics of the study population. A total 459 occlusions were performed (see Additional file 2). In 19 patients, the measurements could not be obtained at all four time points due to various reasons: extubation (*n* = 7), agitation (*n* = 2), low EAdi (*n* = 3), return to controlled mode (n = 2), death (*n* = 1) and others (*n* = 4).

**Table 1:** Main characteristics of the study population

Characteristics	*N* = 31
Age (years), median [IQR]	69 [55.5–72]
Sex, male/female	22/9
BMI (kg/m^2^), median [IQR]	24.7 [21.6–26]
Comorbidity, *n* (%)	
Cardiac diseases	9 (29%)
Diabetes mellitus	6 (19%)
COPD	4 (13%)
Reason for admission, *n* (%)	
Pneumonia	13 (42%)
Postoperative	8 (26%)
Trauma	7 (23%)
Others	3 (10%)
ARDS at admission, *n* (%)	9 (29%)
Sepsis during admission, *n* (%)	6 (19%)
Duration of MV on *T* = 0 (days), median [IQR]	10 [8.5–18.5]
Partially supported mode before *T* = 0	9 [4–14]
Controlled mode before *T* = 0	1 [0–3.5]
Total days of MV (days), median [IQR]	24 [14.5–29.5]
NAVA level, median [IQR]	0.7 [0.5–1.2]
Tidal volume (ml), median [IQR]	450 [381–554]
Respiratory rate (per minute), median [IQR]	25 [18–30]
PEEP (cmH_2_O), median [IQR]	8 [6–10]
Use of opioids/sedatives, *n* (%)	16 (51.6%)
Total LOS ICU (days), median [IQR]	26 [20–34]
Total LOS hospital (days), median [IQR]	41 [23–52.5]
Died within the study period, *n* (%)	1 (3%)

*Abbreviations: ARDS* acute respiratory distress syndrome, *BMI* body mass index, *COPD* chronic obstructive pulmonary disease, *ICU* intensive care unit, *IQR* interquartile range, *LOS* length of stay, *MV* mechanical ventilation, *NAVA* neurally adjusted ventilatory assist, *PEEP* positive end-expiratory pressure

### NMEoccl variability

**Fig. 2:**
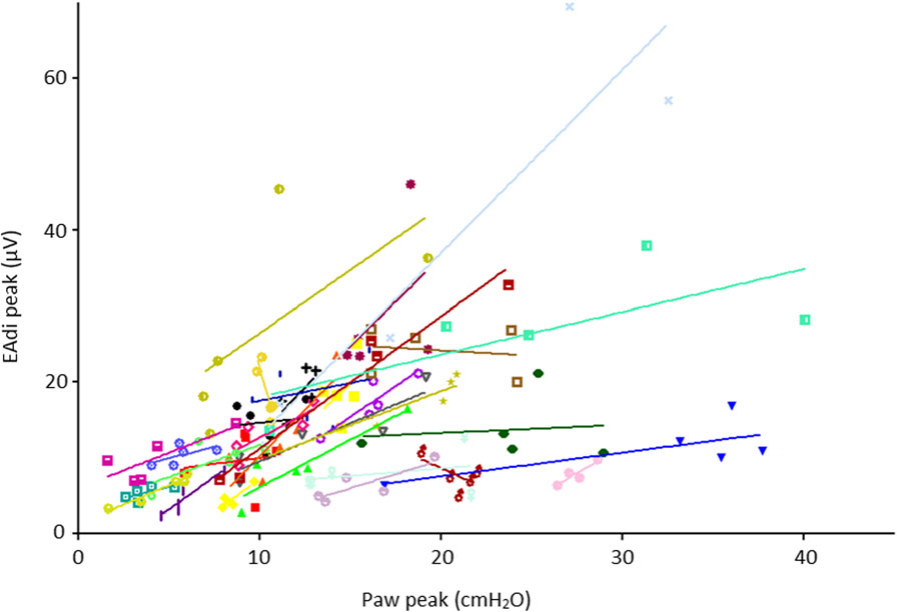
Overview of the correlation of airway pressure (Paw) peak and electrical activity of the diaphragm (EAdi) peak of all maneuvers at time *T* = 0. Each color represents an individual patient with five repeated measurements (dots) and the corresponding slope (line)

A representative NMEoccl maneuver is shown in Fig. 1. At *T* = 0, 149 maneuvers were obtained in 31 patients, 6 maneuvers were lost due to technical issues. Mean ∆Paw was 14.1 ± 7.9 cmH_2_O and mean ∆EAdi 14.8 ± 9.9 μV. Mean NMEoccl was 1.22 ± 0.86 cmH_2_O/μV (ranging from 0.41 to 3.56 cmH_2_O/μV), with a RC of 82.6%. This implies that when NMEoccl is 1.0 cmH_2_O/μV, it is expected with a probability of 95% that the subsequent measured NMEoccl will be between 0.17 and 1.83 cmH_2_O/μV. When the EAdi of this patient during normal breathing is 10 μV, the estimated pressure generated by the inspiratory muscles would be somewhere between 1.1 cmH_2_O and 12.2 cmH_2_O (calculated as EAdi * NMEoccl/1.5, see “Methods”). In addition, the RC of NMEoccl calculated by the AUC and at fixed points on the EAdi curve (Fig. 2) remained high: 87.7% for AUC and 85.5–175.9% at fixed points. The RC of the separate components of NMEoccl was 95.7% for EAdi and 73.9% for Paw. Figure 2 shows there was moderate correlation between ∆EAdi and ∆Paw in the individual patient (mean *r* = 0.52, *p* < 0.0005; ranging from *r* = − 0.90 to *r* = 1.0).

**Fig. 3:**
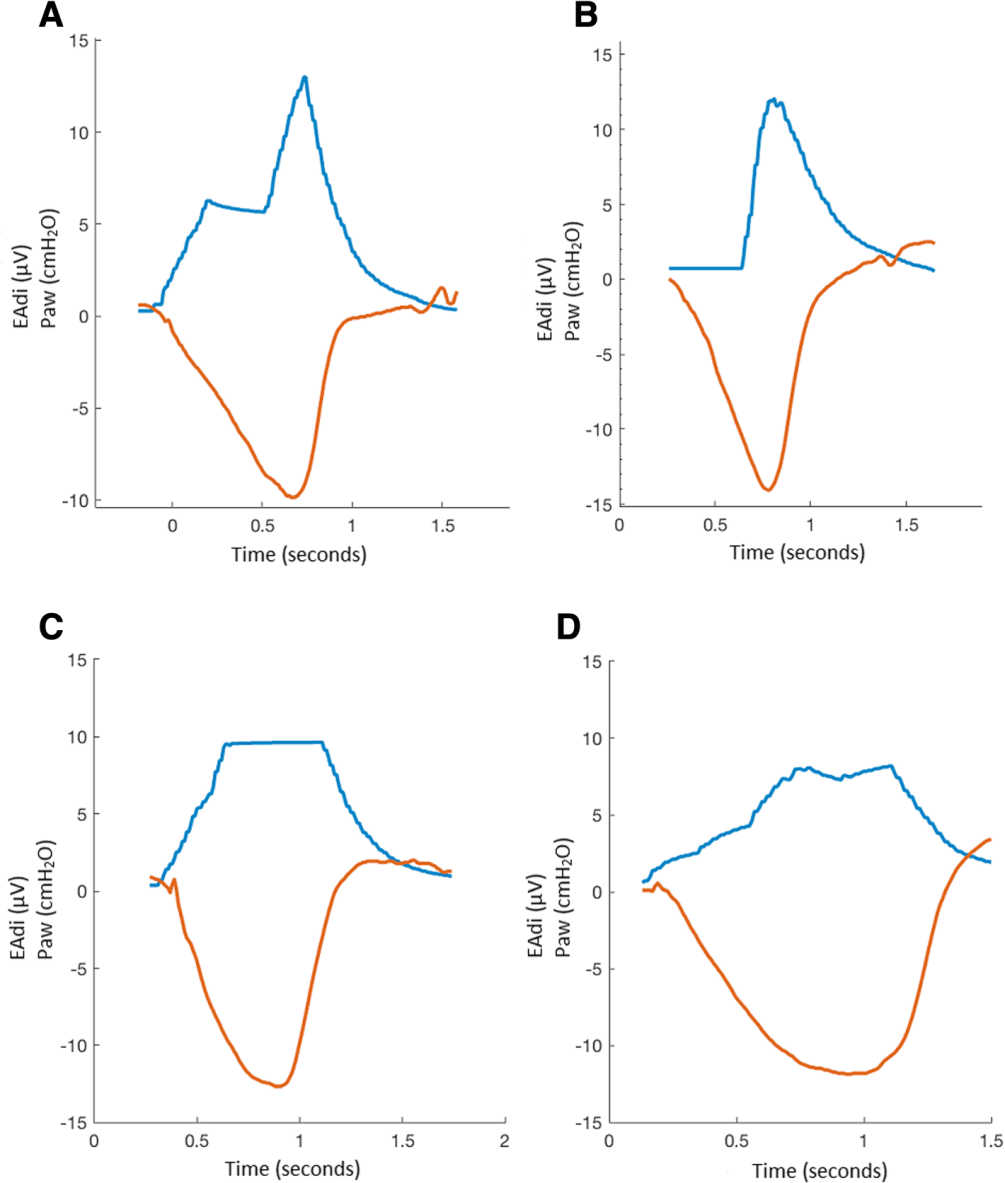
Four examples of electrical activity of the diaphragm (EAdi) waveform irregularities during an end-expiratory occlusion. The blue line represents the EAdi signal expressed in microvolts. The orange line represent the airway pressure (Paw) expressed in centimeters of water. **a** Slope < 0 during the ascending part of the EAdi waveform. **b** Delay in start of EAdi peak. **c** EAdi peak cut off. **d** Split EAdi peak

Upon visual inspection it appeared that some of the EAdi tracings exhibit a rather non-physiological shape: a plateau during inspiration (while an increase would be expected) or at maximum inspiration, or a delayed increase in EAdi relative to the decrease in Paw (Fig. 3). It was reasoned that non-physiological-appearing EAdi waveforms contribute to the high NMEoccl variability. Additional file 2 shows every occlusion maneuver obtained in this study. Different mathematical approaches were used in order to try to objectively detect and exclude non-physiological EAdi waveforms (See Additional file 3). Despite these mathematical approaches the NMEoccl repeatability remained high with a RC of 63.4%.

Finally, we pragmatically selected three out of five occlusions with the lowest variability and averaged the three values to obtain a single NMEoccl for each individual patient. This approach reduces the influence of erroneous values, irrespective of the origin, and will result in a more reproducible NMEoccl value. As a result, the RC improved to 29.8%. This approach was used for subsequent analysis.

### Correlation of NMEoccl variability with clinical parameters and inspiratory time

The median CoV NMEoccl at *T* = 0 was 23.1% (IQR 18.7–29.9%). After dividing the study population into two groups with CoV higher or lower than median, respectively, no significant differences in clinical parameters were found (see Additional file 4). Median Ti during occlusion was 0.51 s (IQR 0.35–0.65 s) and of the preceding breaths 0.65 s (IQR 0.48–0.84 s). On average, in 48% of the measurements within a patient, Ti of the occluded breath was longer compared to Ti of the preceding breaths. Only moderate negative correlation was found between Ti and NMEoccl (*r* = − 0.219).

### Changes in NMEoccl over time

Twelve patients completed the 72 h study period. In these patients, NMEoccl at *T* = 0 was 0.8 cmH_2_O/μV (IQR 0.7–1.1 cmH_2_O/μV) and did not change over time (*p* = 0.75); 0.7 cmH_2_O/μV (IQR 0.4–0.9 cmH_2_O/μV), 0.8 cmH_2_O/μV (IQR 0.6–1.1 cmH_2_O/μV), 0.9 cmH_2_O/μV (IQR 0.6–1.3 cmH_2_O/μV) for *T* = 12, *T* = 24 and *T* = 72, respectively.

### Diaphragm muscle effort

The mean EAdi of five unloaded breaths before the end-expiratory occlusion were used to calculate Pmus. At *T* = 0, median Pmus was 9.4 cmH_2_O (IQR 6.0–12.8 cmH_2_O) and did not change over time (*p* = 0.58); 10.4 cmH_2_O (IQR 5.4–15.4 cmH_2_O), 10.7 cmH_2_O (IQR 4.8–13.1 cmH_2_O) and 8.2 cmH_2_O (IQR 6.0–15.9 cmH_2_O) for *T* = 12, T = 24 and *T* = 72, respectively. The median Pmus varied widely among patients, ranging from 1.8 to 36.0 cmH_2_O. Based on previous literature [21, 23], we defined a physiological Pmus between 5 and 10 cmH_2_O. At *T* = 0, 6 patients (19.4%) had a mean Pmus < 5cmH_2_O and 12 patients (38.7%) > 10cmH_2_O, indicating ventilator over-assist or under-assist, respectively.

**Fig. 4:**
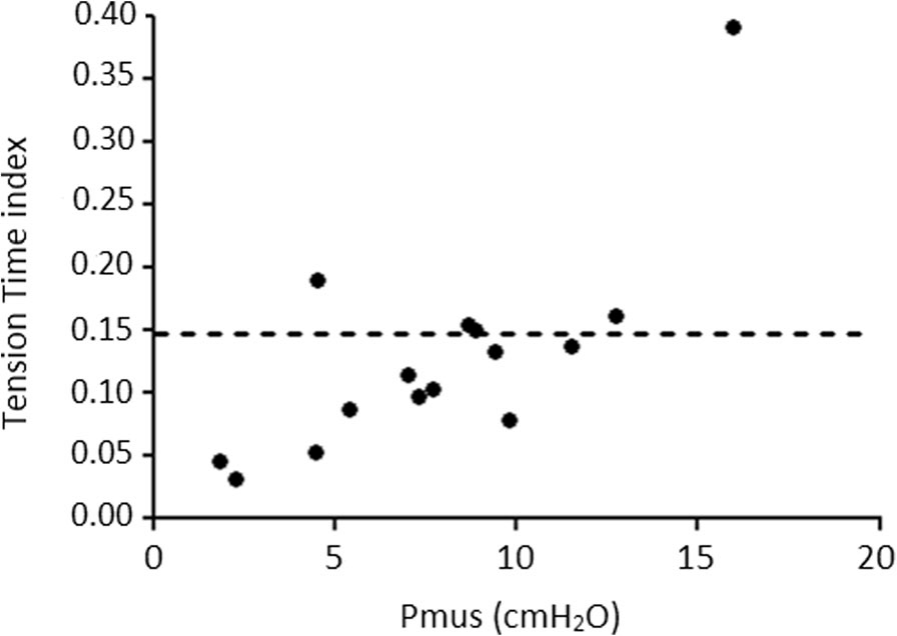
Overview of correlation between the tension-time index and inspiratory pressure (Pmus) in 15 patients in whom maximum inspiratory pressure was measured (dots). The dotted line represents the cut off for diaphragm fatigue [36]

MIP was obtained in 15 patients in the week before or after the NME measurements, which allowed us to calculate TTI. Median MIP was 29 cmH_2_O (IQR 24–38 cmH_2_O) and median TTI was 0.12 (IQR 0.08–0.17). Figure 4 shows the relationship between TTI and Pmus.

## Discussion

The main findings of the present study can be summarized as follows: (1) repeated measurements of NMEoccl within an individual patient exhibited unacceptably high variation, indicating that a single NMEoccl cannot be used to estimate pressure generated by the inspiratory muscles; (2) no correlation was found between NMEoccl variability and clinical parameters; (3) extensive waveform analyses did not improve the repeatability of NMEoccl; (4) NMEoccl and Pmus remain stable over time in a heterogeneous group of patients and (5) both low and high diaphragm effort are common in this cohort of patients on partially supported mode.

### NMEoccl variability

Variability of both components of NMEoccl (Paw and EAdi) is expected given the variability in inspiratory drive, even during occlusions. However, NMEoccl itself is independent from respiratory drive and should therefore be more stable from breath to breath.

Beck and colleagues reported a linear relationship between EAdi and Pdi in healthy subjects, at least up to 75% of maximum force [32]. Their subsequent study also demonstrated a linear relationship between EAdi and Pdi in patients with acute respiratory failure [20]. Bellani et al. showed that NMEoccl derived from airway pressure closely reflects NME during normal breathing (NMEdyn) derived from Pes and concluded that calculation of NMEoccl allows a clinically valuable estimate of inspiratory effort [21]. In this latter study, two end-expiratory occlusions were obtained in each patient, but repeatability was not reported. Furthermore, they report that despite changes in level of support, NMEoccl remained rather stable within individual patients, as supported by the linear relationship between Pmus and EAdi (*r*^2^ = 0.78) [21]. This is in apparent contrast with our findings, as we found only moderate correlation between Pmus and EAdi (Fig. 2). However, differences in data analysis should be acknowledged. Bellani used the Pearson correlation coefficient to test the correlation between Paw and EAdi, in which all measurements from different patients were analyzed together as if they were from a single patient. However, the variability of *between-subject* measurements is different compared to the variability of the *within-subject* measurements [33]. Therefore, calculating the correlation coefficient of repeated observations, as in the current study, seems more appropriate [30, 31]. Indeed, Fig. 2 demonstrates that the slope of Paw and EAdi in the individual patient is highly variable. This is consistent with the results of Bellani, exhibiting high inter-individual variability [21].

The high NMEoccl repeatability reported in the current study precludes its application in clinical practice. Based on visual inspection of the EAdi waveforms, we proposed that suboptimal filtering and replacement of cardiac electric activity by the ventilator software are important. Several techniques for waveform analyses were applied, but did not improve repeatability, suggesting that in addition to suboptimal filtering other factors might be involved. Beck et al. showed that an increase in volume from functional reserve capacity (FRC) to total lung capacity (TLC) reduces Pdi by 60% for a given EAdi [34]. Similarly, muscle weakness may affect NMEoccl, but both are unlikely to explain the high variability in our study, given that all measurements were obtained in a time window of 5–10 min.

As none of the techniques for waveform analyses resulted in improved NMEoccl repeatability, a more pragmatic approach was explored that is also feasible in clinical practice. In thermodilution cardiac output measurements, three repeated measurements are averaged, provided that these values are within 10% of their average. If not, a total of five measurements are performed in which the lowest and highest value are eliminated [35]. In our study, this strategy reduced the influence of erroneous NMEoccl values, irrespective of the cause, and will result in a more reproducible value of NMEoccl.

### NMEoccl as monitoring tool

Theoretically, the NMEoccl could be helpful to titrate ventilator support in patients on partially supported modes. NMEoccl is calculated by dividing ∆Paw by ∆EAdi during an end-expiratory occlusion. During an occlusion ∆Paw equals ∆Pmus and therefore NMEoccl can be obtained without direct measurement of Pmus (requiring an esophageal balloon). Rearranging this formula to Pmus = NMEoccl * EAdi allows calculation of Pmus breath by breath, after dividing this value by 1.5 to correct for differences in NME obtained under static and dynamic conditions [21]. Interestingly, NMEoccl may be used to evaluate respiratory muscle function over time. A decrease in NMEoccl indicates that the respiratory muscles are less efficient in converting electrical activity into pressure. Possible causes for variability in NMEoccl require further studies, but may include intrinsic positive end-expiratory pressure (PEEP) and impaired function of the contractile proteins.

In our study population there were no significant changes in NMEoccl over time (*p* = 0.75), which corresponds to the results of Bellani et al. [23]. However, it should be noted that during our study period the ventilatory settings were not fixed, which might explain why NMEoccl did not change over time.

In our study Pmus varied among patients but remained relatively stable in individual patients over time. In some patients estimated inspiratory effort was > 20 cmH_2_O. An important question is whether a relatively high inspiratory pressure generated by the respiratory muscles may result in the development of contractile fatigue. A TTI ≥ 0.15 puts the diaphragm at risk of development of fatigue [36]. In our study, all patients except for two, with a Pmus < 12 cmH_2_O, had a TTI < 0.15. This might suggest that titration of ventilatory support to a pressure < 12 cmH_2_O could limit the risk of fatigue development. However, this has to be studied before it can be applied in clinical practice.

### Strengths and limitations

The strengths of our study are the high number of occlusions analyzed and the fact that at each time point five repeated occlusions were obtained. This allows thorough analysis of the repeatability of NMEoccl and provides methods to improve its variability under clinical conditions. In addition, several waveform analysis techniques were performed to evaluate the high NMEoccl variability; however, this did not improve the RC for NMEoccl. It was suggested that suboptimal filtering might be important. Software engineers should further improve ventilator software for EAdi signal filtering.

Several limitations should be acknowledged. First, patients in our study did not have an esophageal balloon in situ and therefore we could not validate our measurements against the gold standard. However, previous studies have shown excellent correlation between ∆Paw and ∆Pes during an occlusion maneuver [37–39]. Second, our study was conducted in a single center and in a selected group of ICU patients. The generalizability of the findings needs to be assessed.

## Conclusion

End-expiratory occlusion allows measurement of static change in EAdi and Paw for calculation of NMEoccl. This maneuver is simple to conduct and safe in ICU patients ventilated in partially supported mode. However, the present study demonstrates that a single maneuver cannot be used to calculate NMEoccl, given the unacceptably high variability. Further studies should be conducted to improve software for EAdi analysis for this specific purpose. For now, selecting three out of five occlusions with the lowest variability seems to be the best method to estimate inspiratory muscle effort from EAdi.

## Additional files

Additional file 1: NMEoccl calculated as a ratio. Since the variability of NMEoccl increased as the magnitude of the NMEoccl increased, the ratio of a single NMEoccl value to the mean NMEoccl of five repeated measurements was used to calculate the variability of NMEoccl [26]. (A) The difference in NMEoccl is expressed against mean NMEoccl. (B) the ratio of NMEoccl is expressed against mean NMEoccl. (TIF 70 kb)

Additional file 2: Overview of the five repeated occlusions in each individual patient. The blue line represents the EAdi signal expressed in microvolts. The orange line represents the airway pressure (Paw) expressed in cmH_2_O. (DOCX 1942 kb)

Additional file 3: Extensive waveform analyses. This file gives an overview of the extensive waveform analyses that are performed, in order to objectively detect and exclude non-physiological EAdi waveforms. (DOCX 36 kb)

Additional file 4: Correlation of clinical parameters and NMEoccl variability at *T* = 0. First, the coefficient of variation (CoV) was calculated for each patient. The median CoV for NMEoccl at *T* = 0 was 23.1% (IQR 18.7–29.9%). The study population was divided in two groups, with the CoV higher or lower than the median, respectively. CoV = coefficient of variation; COPD = chronic obstructive pulmonary disease; EAdi = electrical activity of the diaphragm; HR = heart rate; IQR = interquartile range; NAVA = neutrally adjusted ventilatory assist; RASS = Richmond agitation sedation scale; RR = respiratory rate; VT = tidal volume. (DOCX 18 kb)

## Abbreviations

ANOVA: Analysis of variance
AUC: Area under the curve
CoV: Coefficient of variation
EAdi: Electrical activity of the diaphragm
EMG: Electromyography
FRC: Functional reserve capacity
ICU: Intensive Care Unit
IQR: Interquartile range
MIP: Maximum inspiratory pressure
NMEdyn: Neuromechanical efficiency index during normal breathing
NMEoccl: Neuromechanical efficiency index (during an end-expiratory occlusion)
Paw: Airway pressure
Pdi: Transdiaphragmatic pressure
Pes: Esophageal pressure
Pmus: Inspiratory pressure
PVBC: Patient ventilator breath contribution
RC: Repeatability coefficient
SD: Standard deviation
Ti: Inspiratory time
TLC: Total lung capacity
TTI: Tension-time index
Ttot: Total respiratory cycle time
Δ: Delta
