# Supplementary figures and images for: Estimation of the diaphragm neuromuscular efficiency index in mechanically ventilated critically ill patients
Source: Crit Care. 2018 Sep 27;22:238. doi: 10.1186/s13054-018-2172-0 (PMC6161422; doi:10.1186/s13054-018-2172-0)

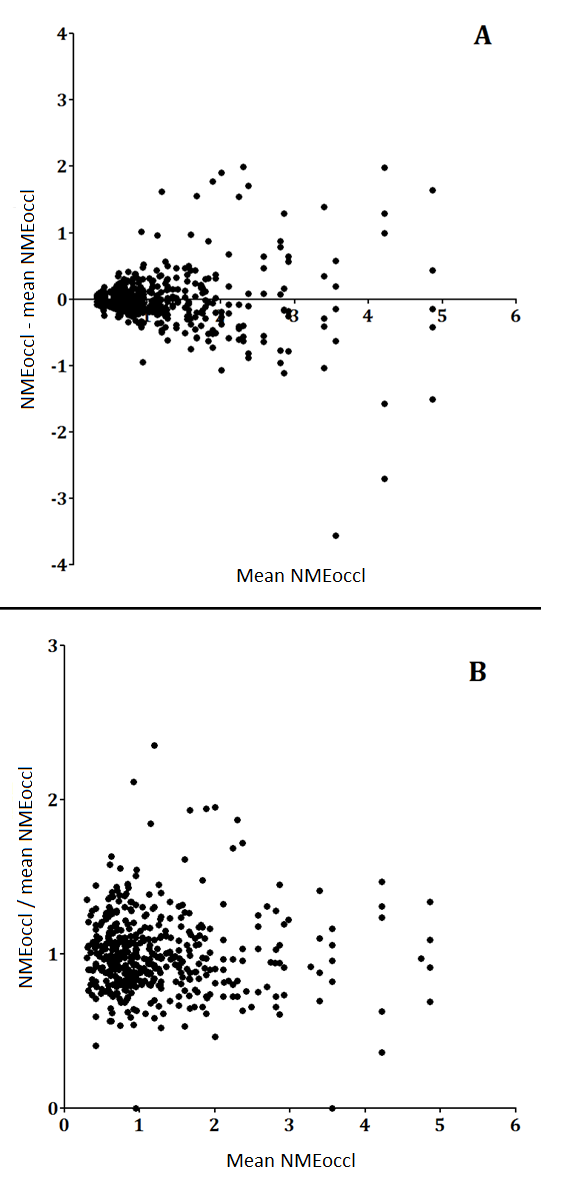

Supplement: Supplementary file 1 — NMEoccl calculated as a ratio. Since the variability of NMEoccl increased as the magnitude of the NMEoccl increased, the ratio of a single NMEoccl value to the mean NMEoccl of five repeated measurements was used to calculate the variability of NMEoccl [26]. (A) The difference in NMEoccl is expressed against mean NMEoccl. (B) the ratio of NMEoccl is expressed against mean NMEoccl. (TIF 70 kb) [file 13054_2018_2172_MOESM1_ESM.tif]

**Additional File 2.**


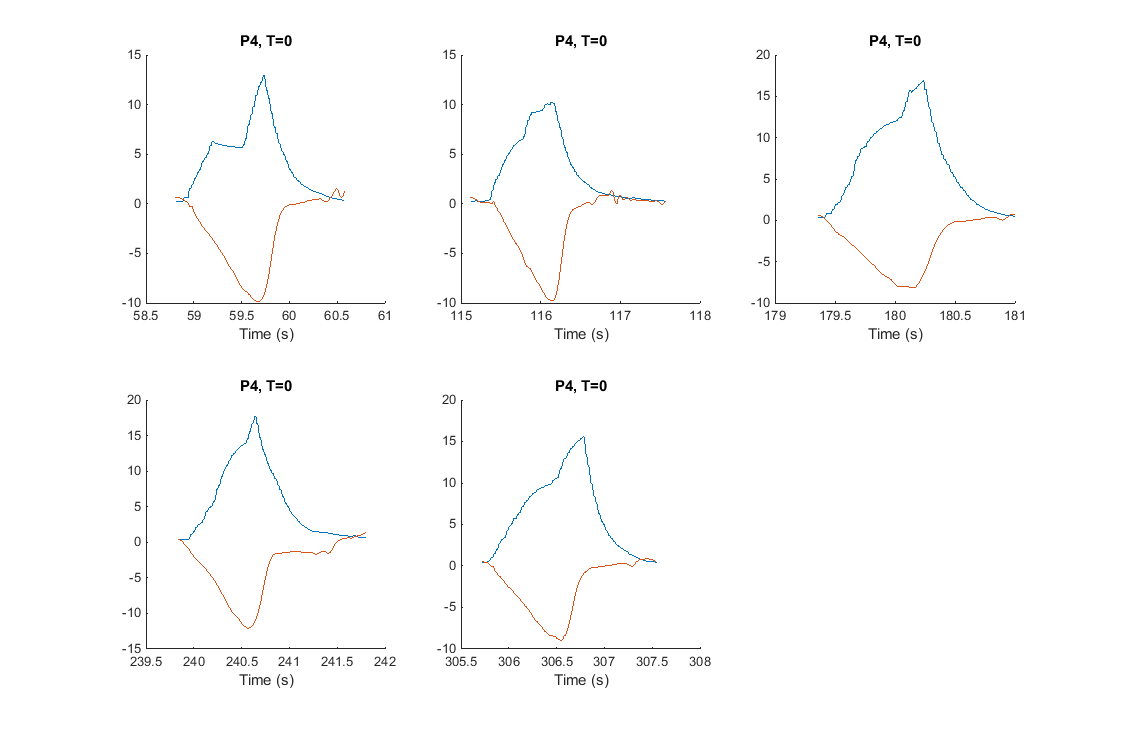

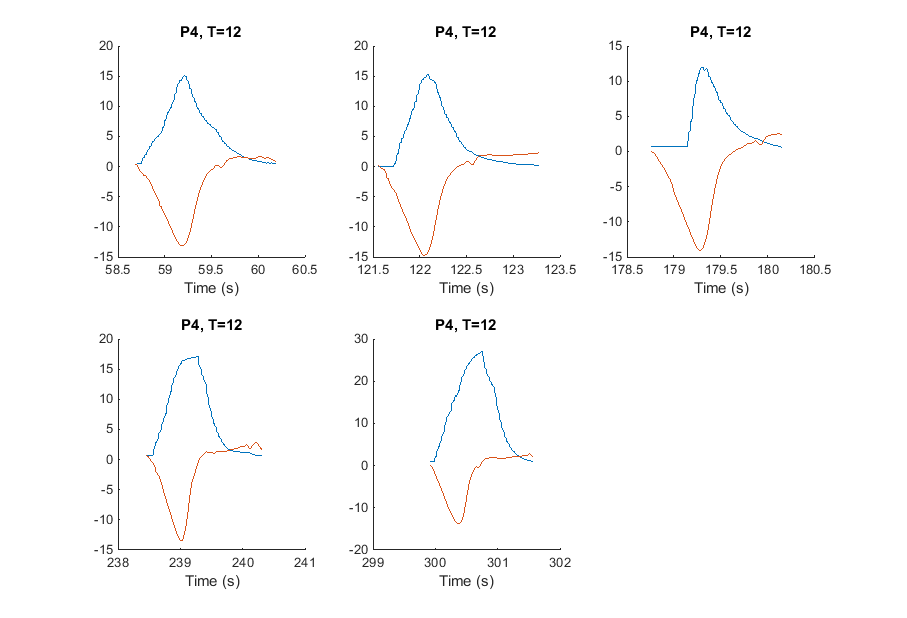


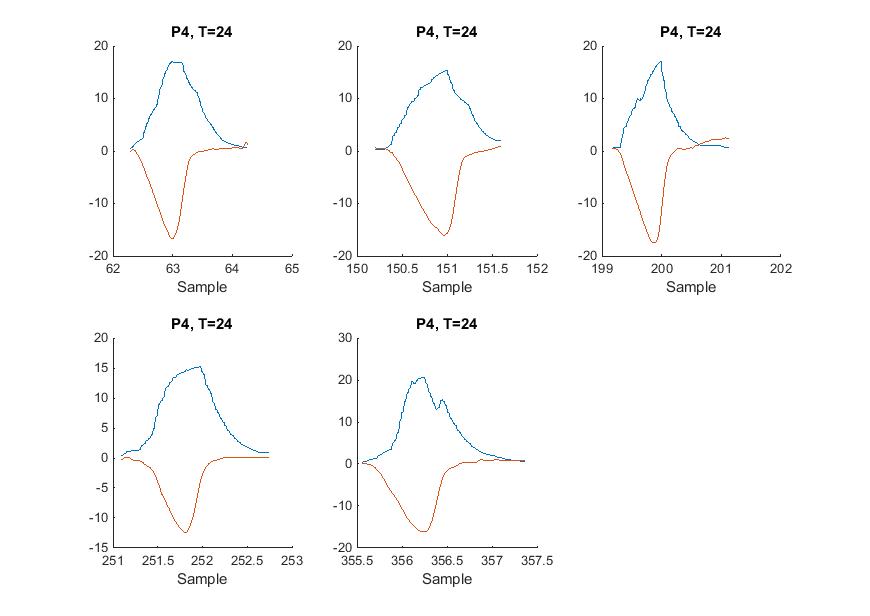

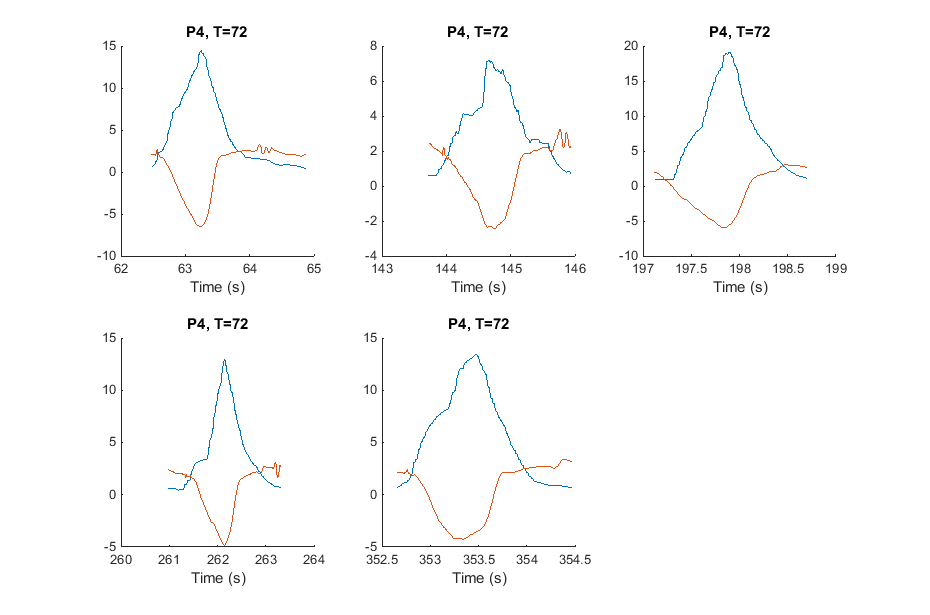


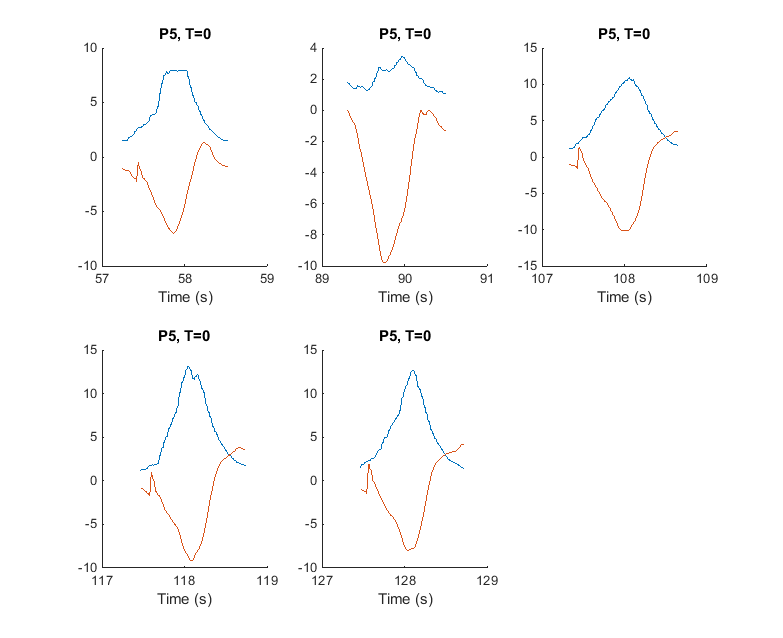

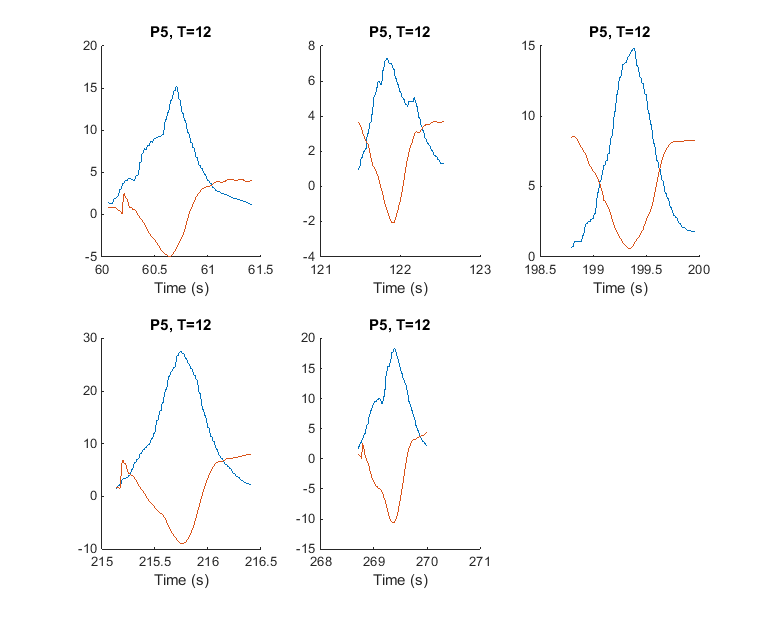


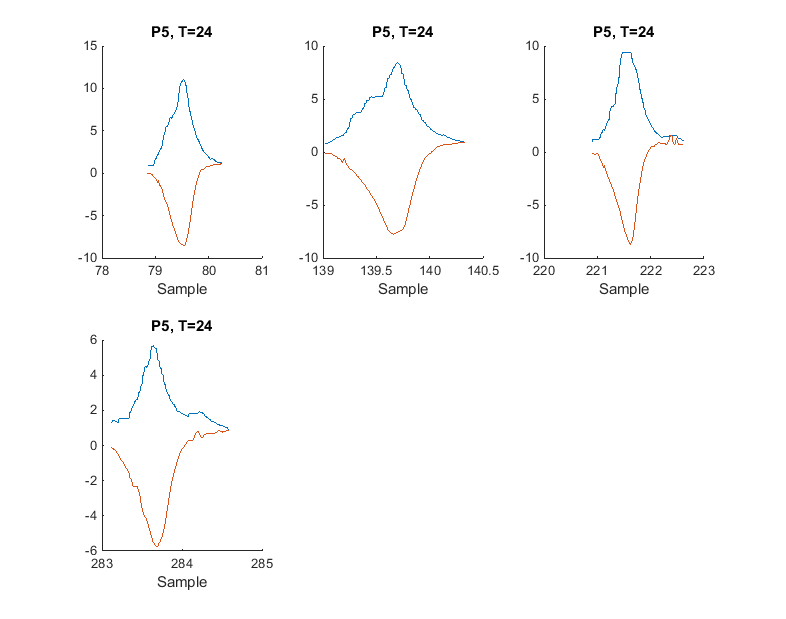

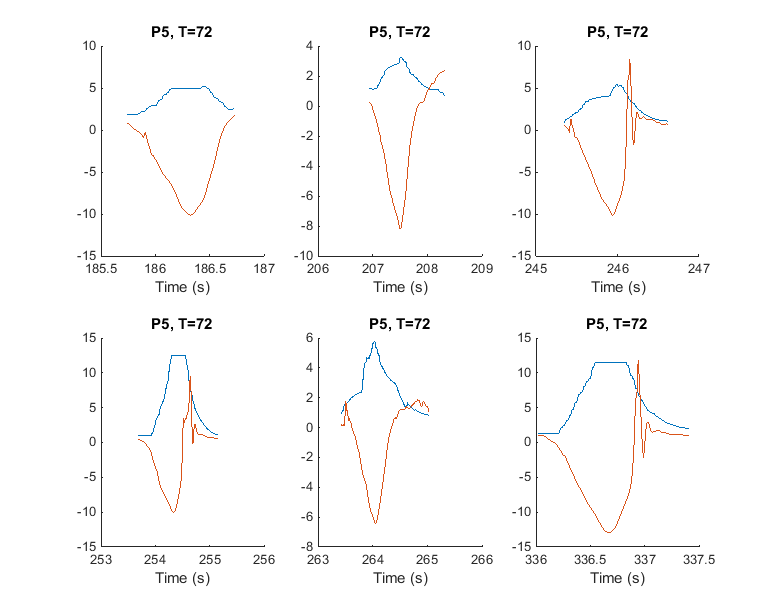


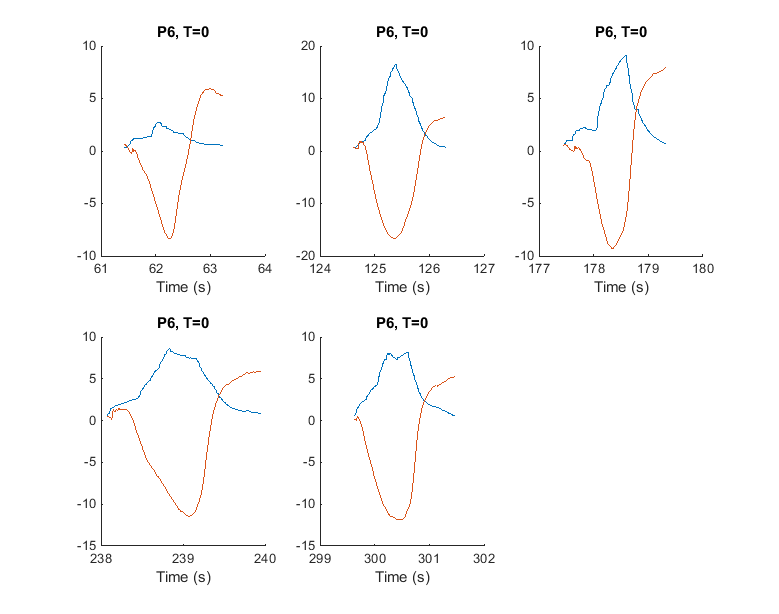

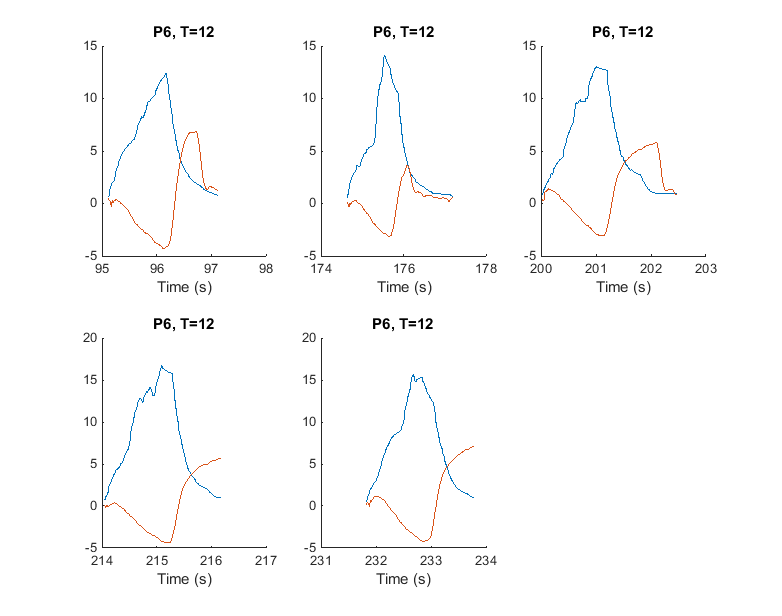


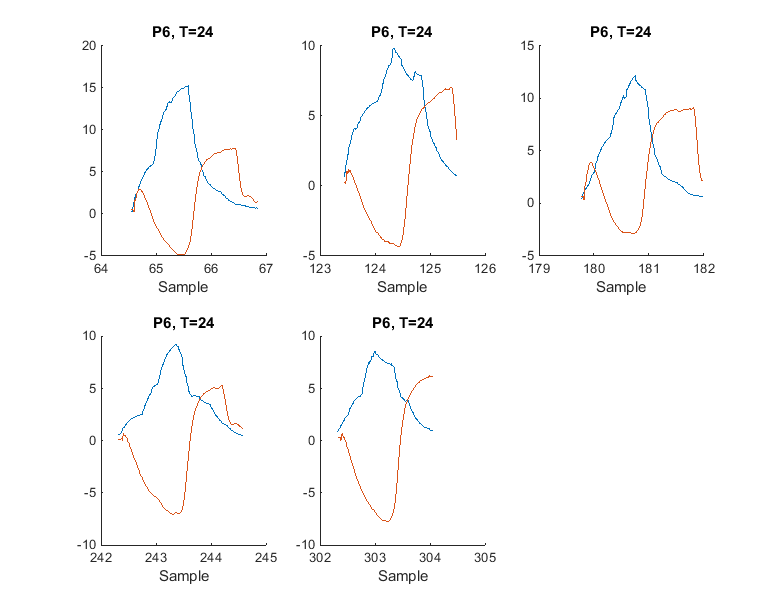


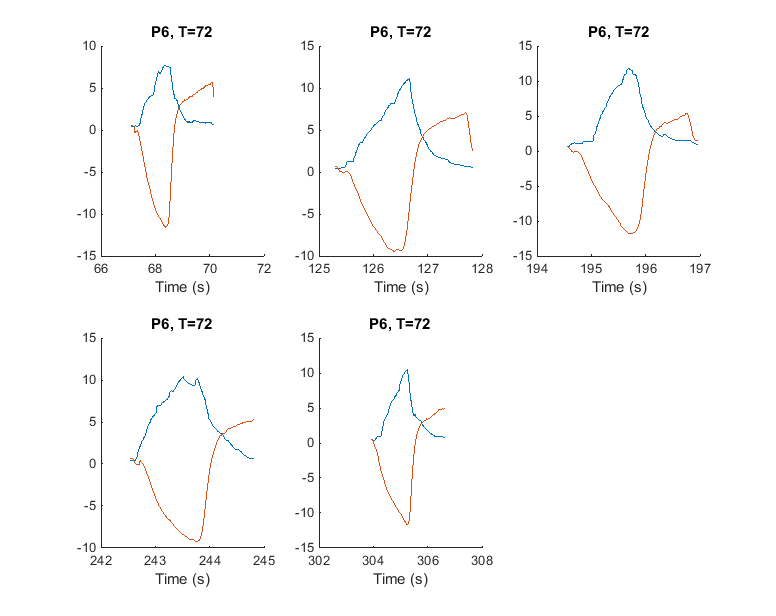


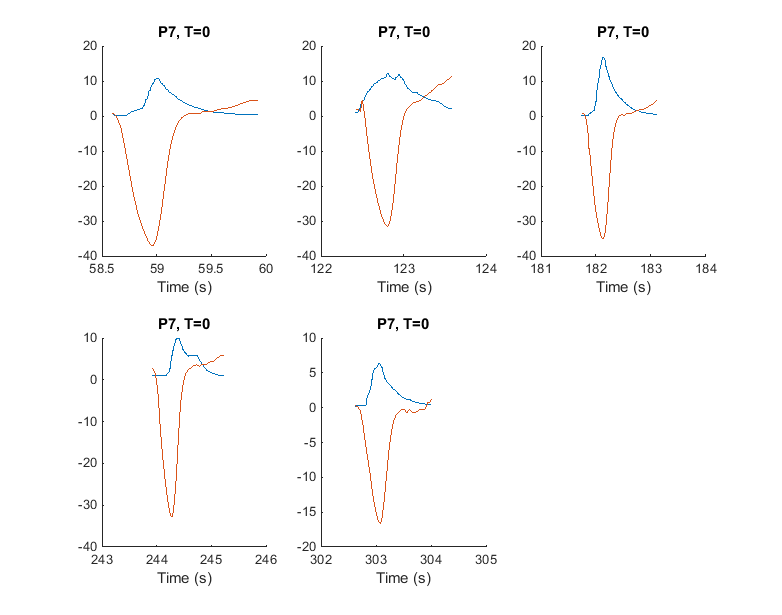


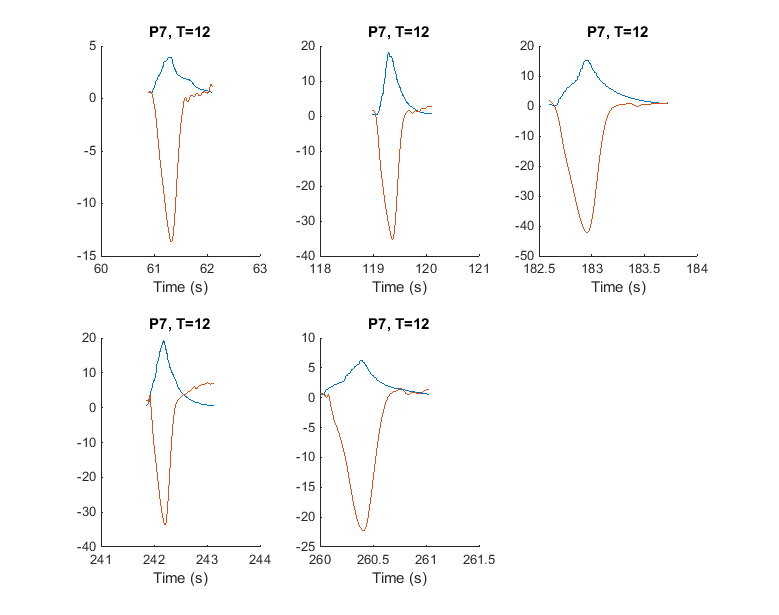


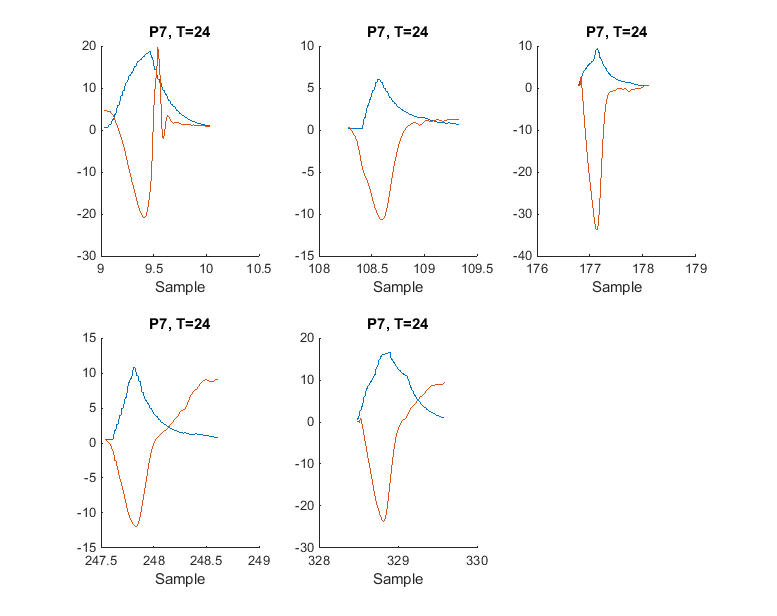


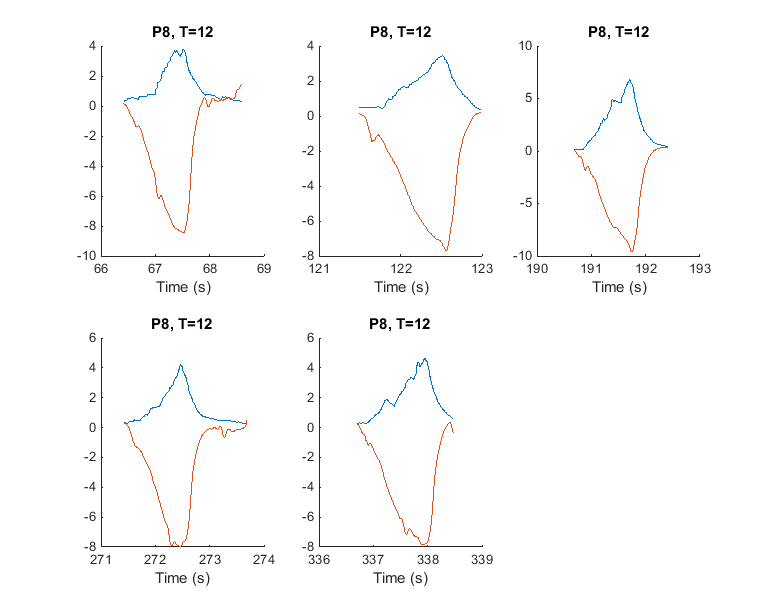

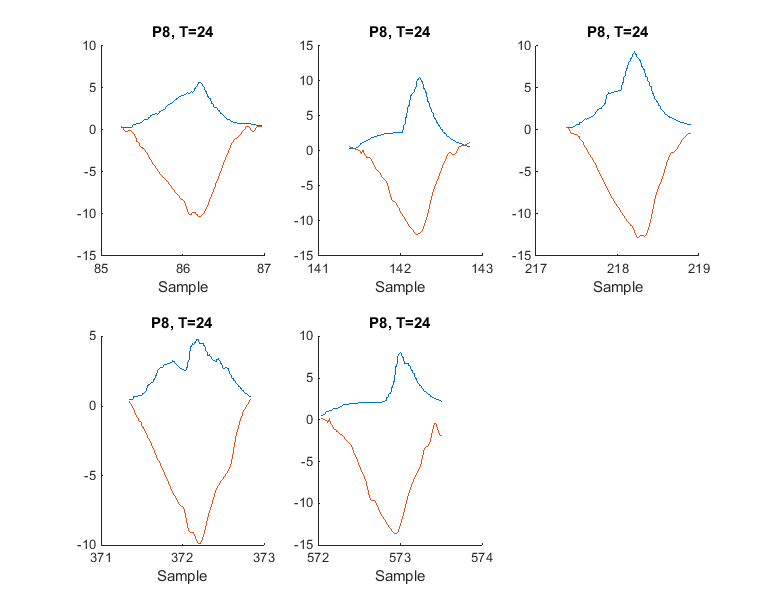


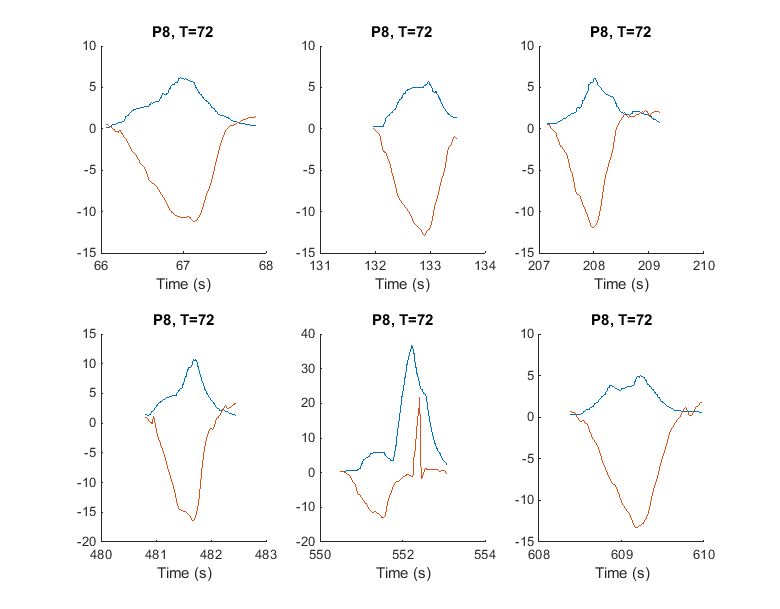


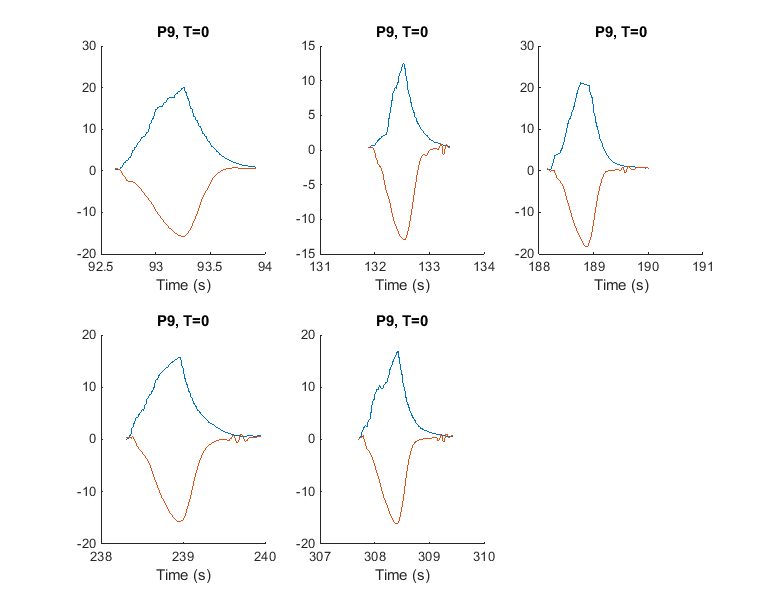


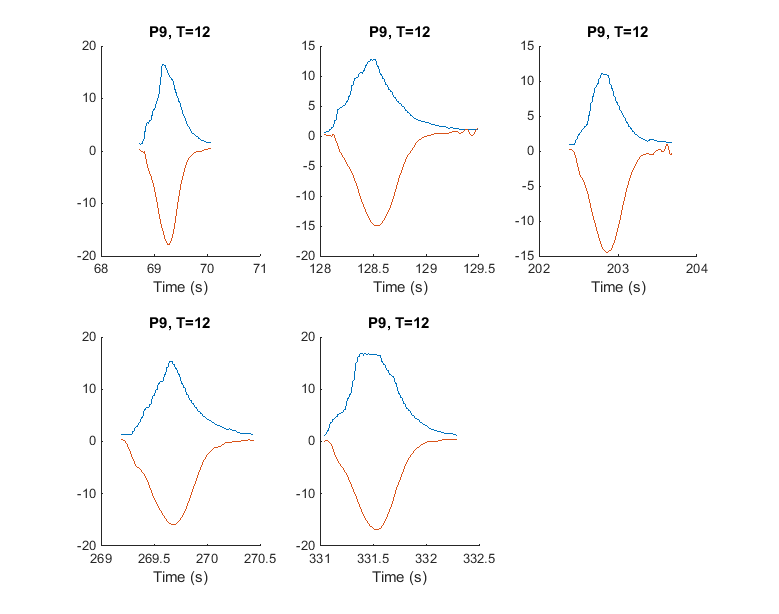


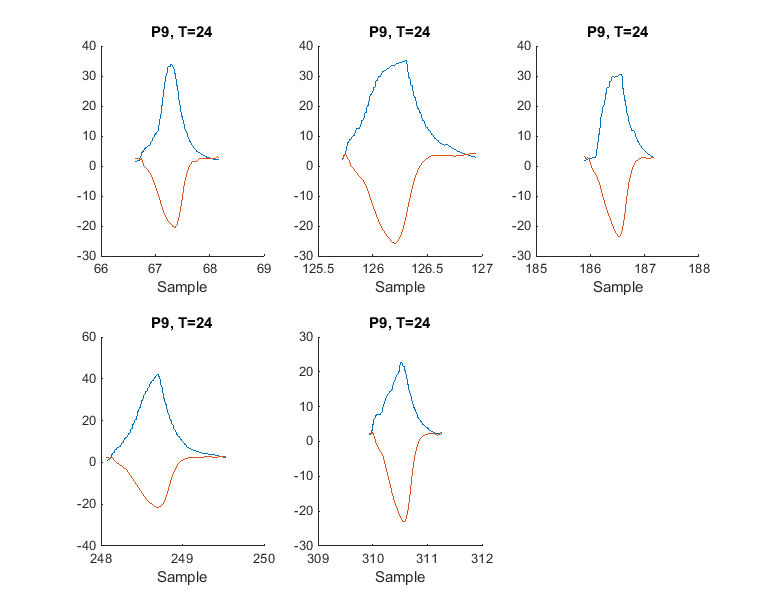


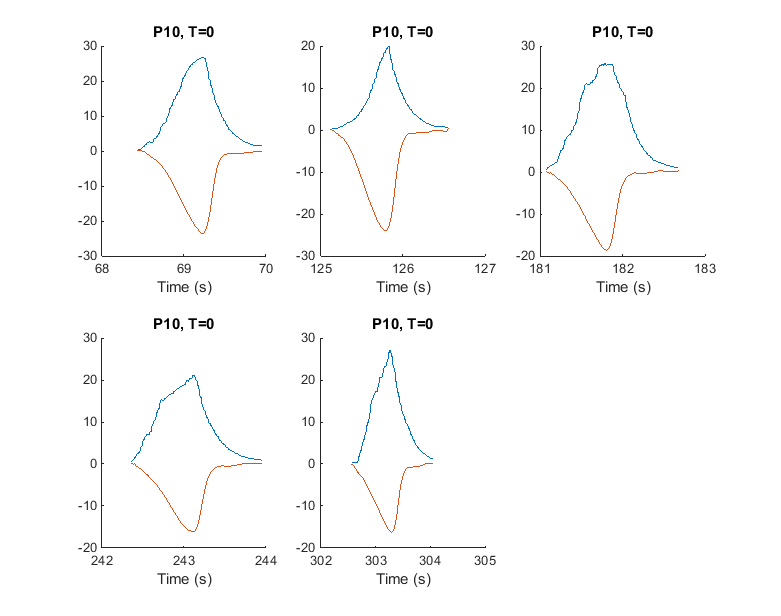


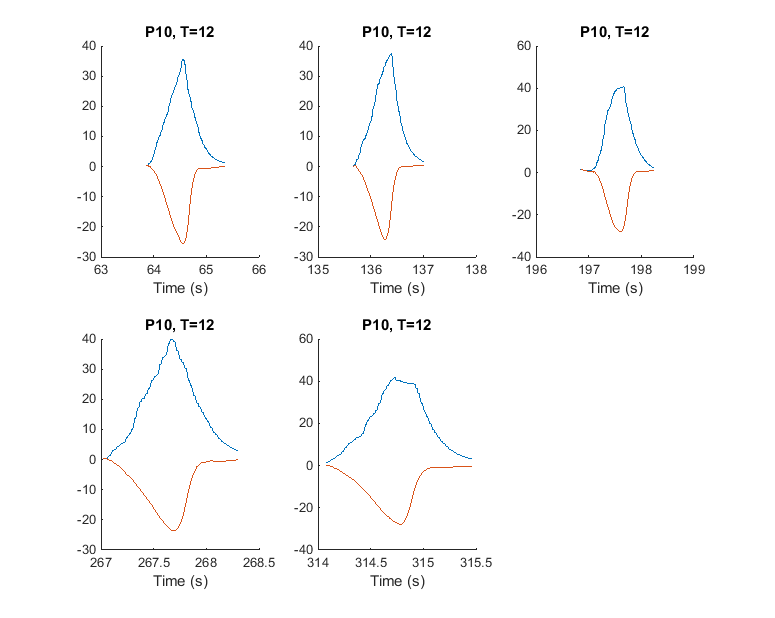


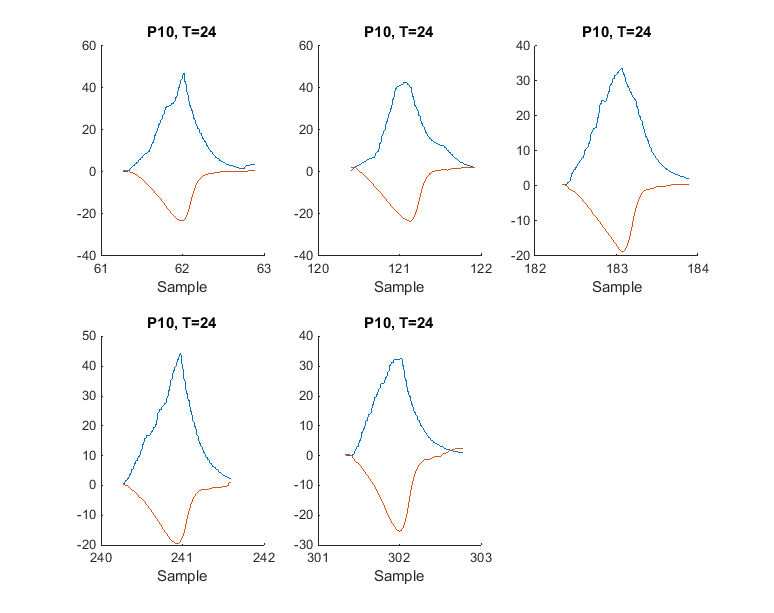

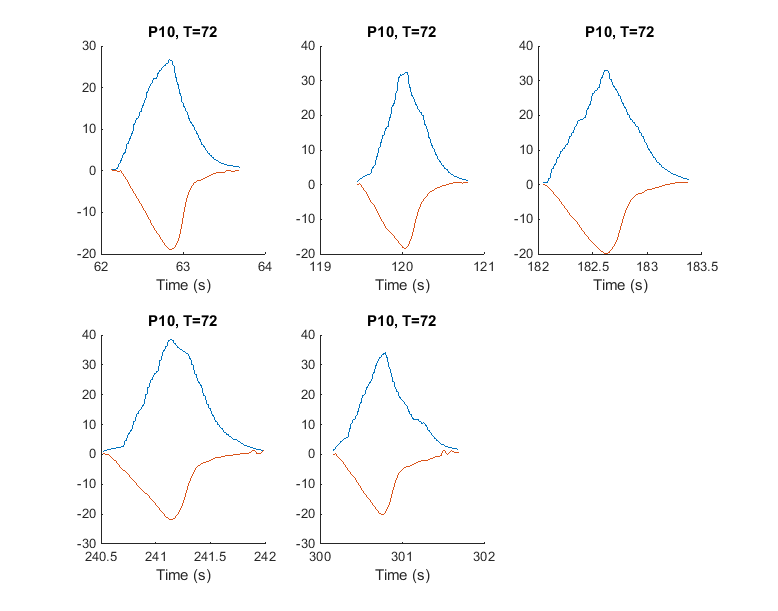


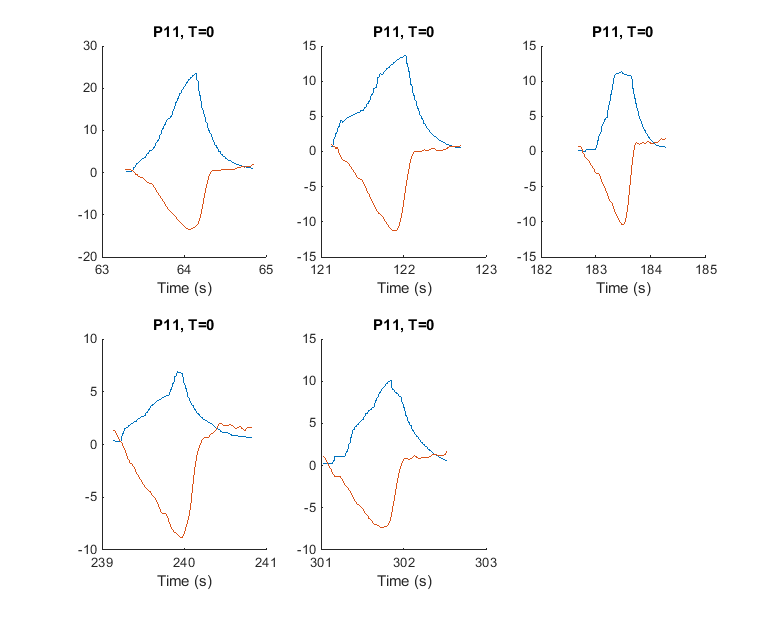


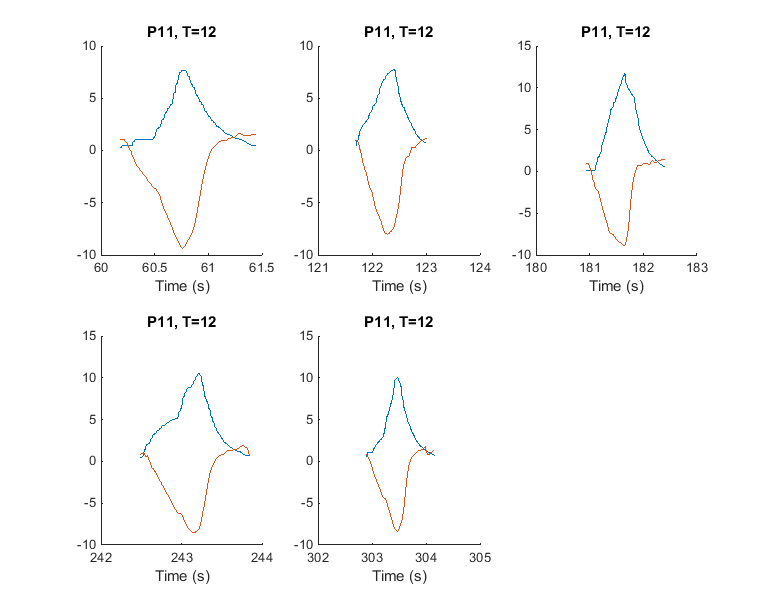


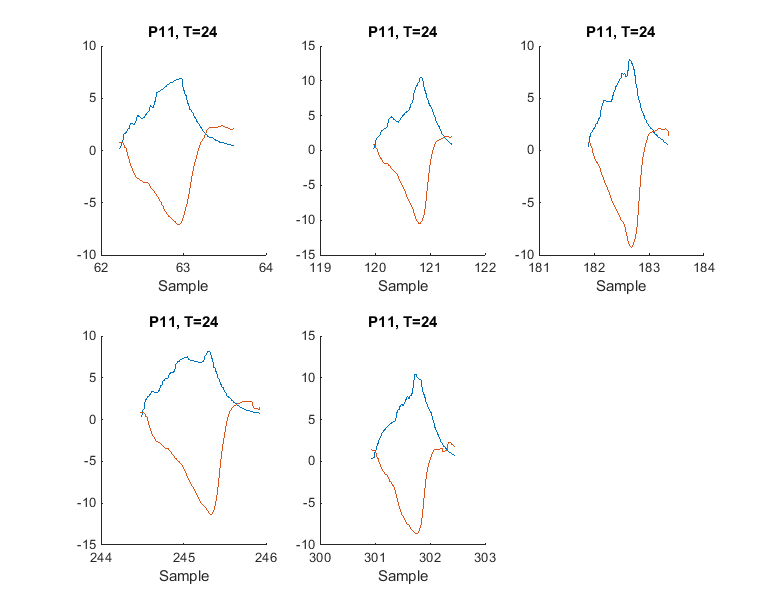


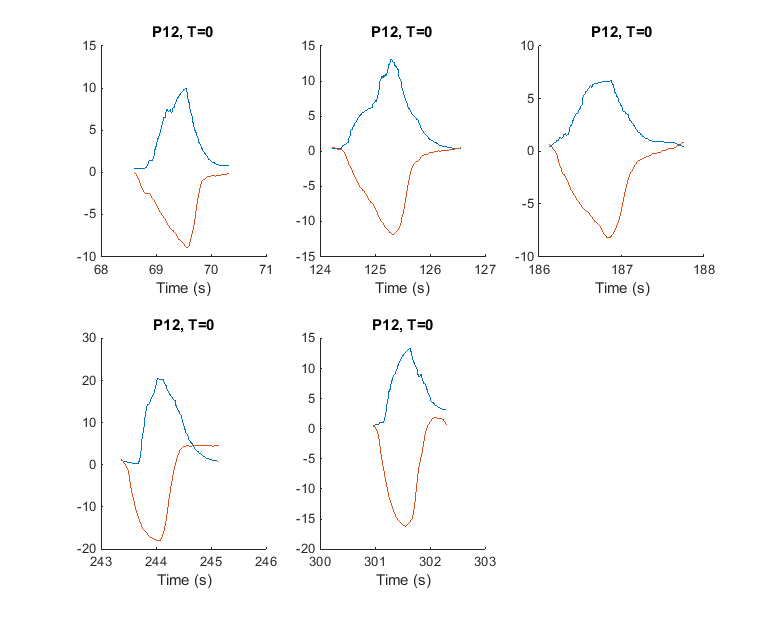

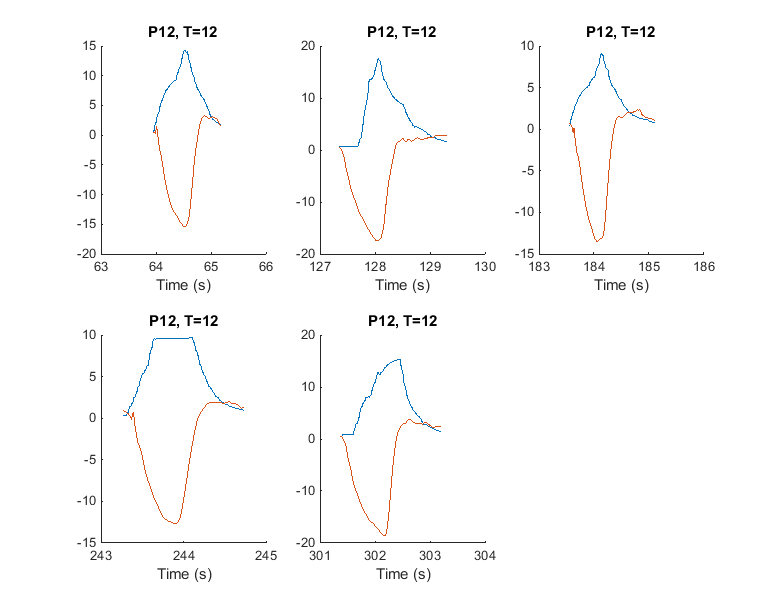


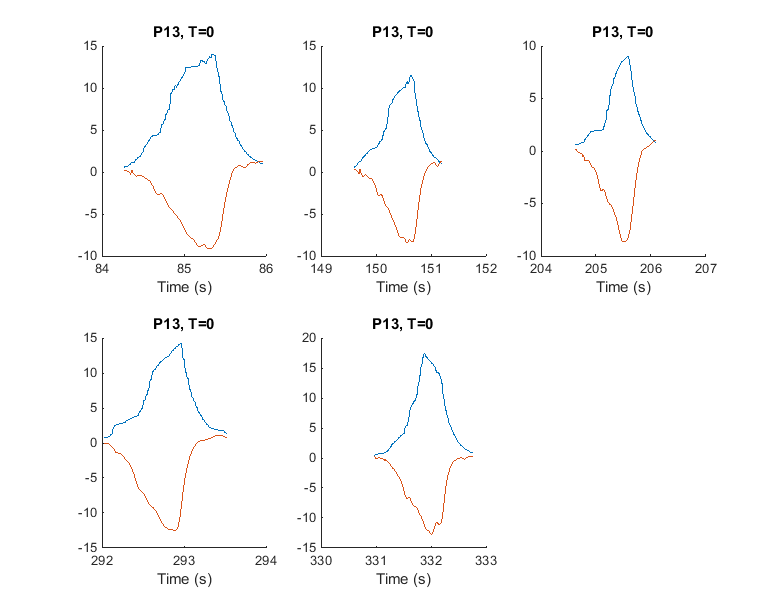


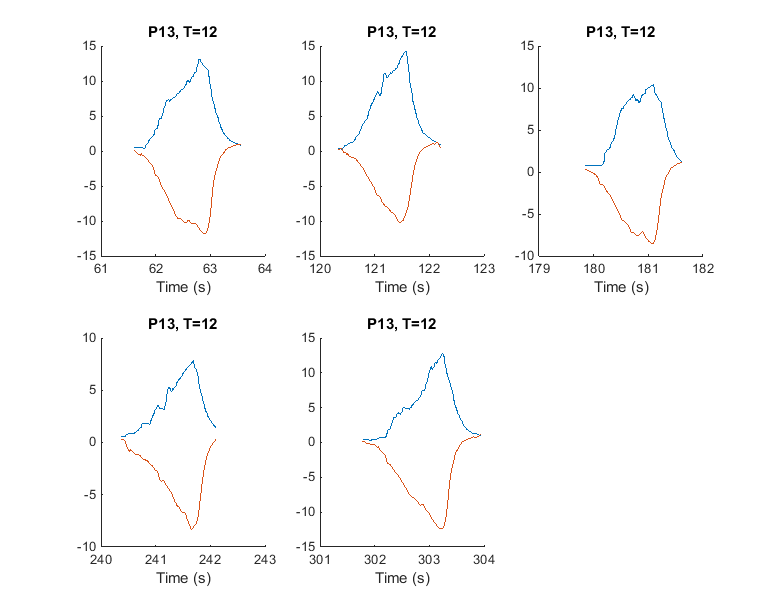


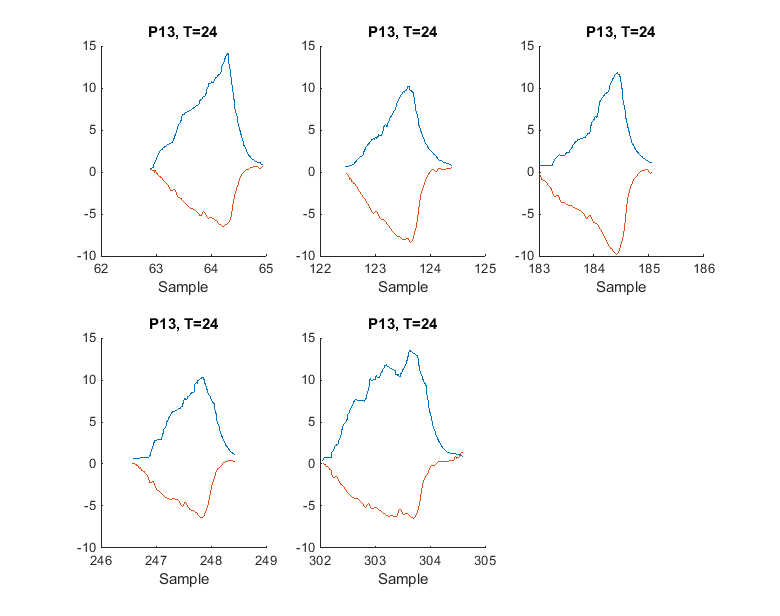


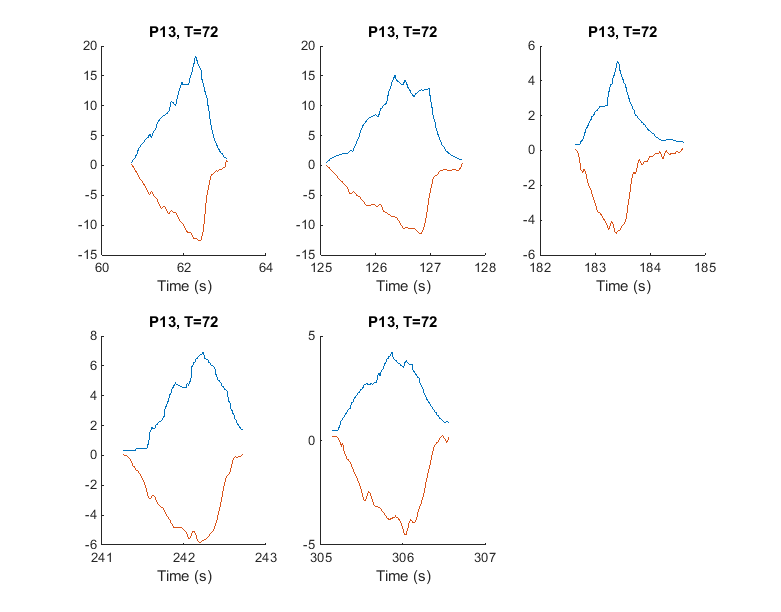


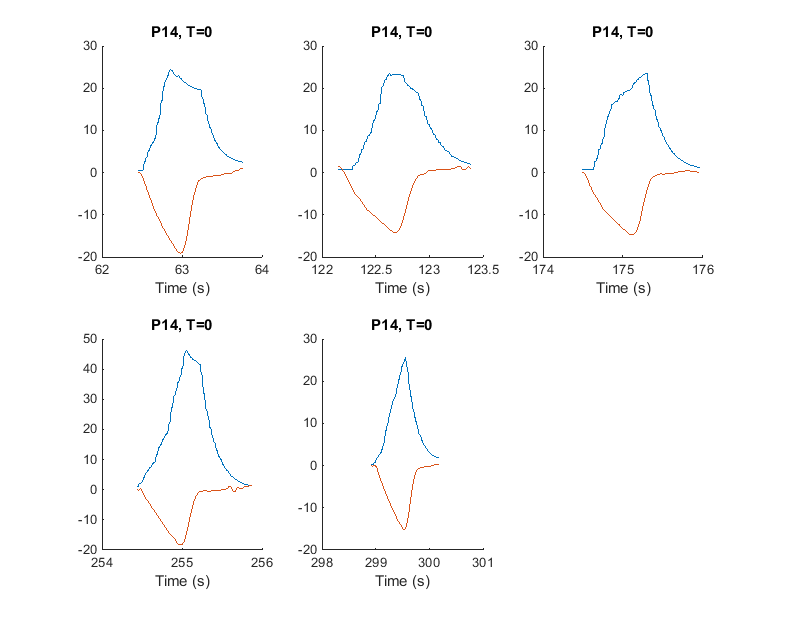


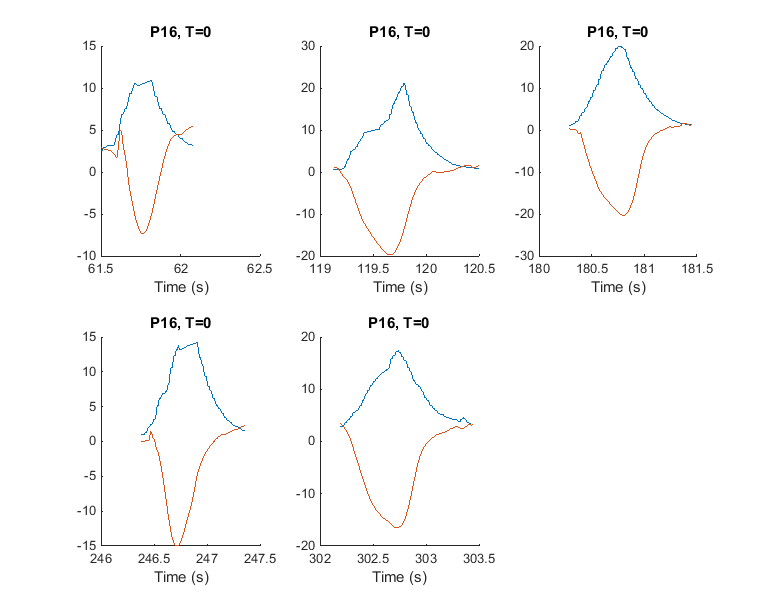


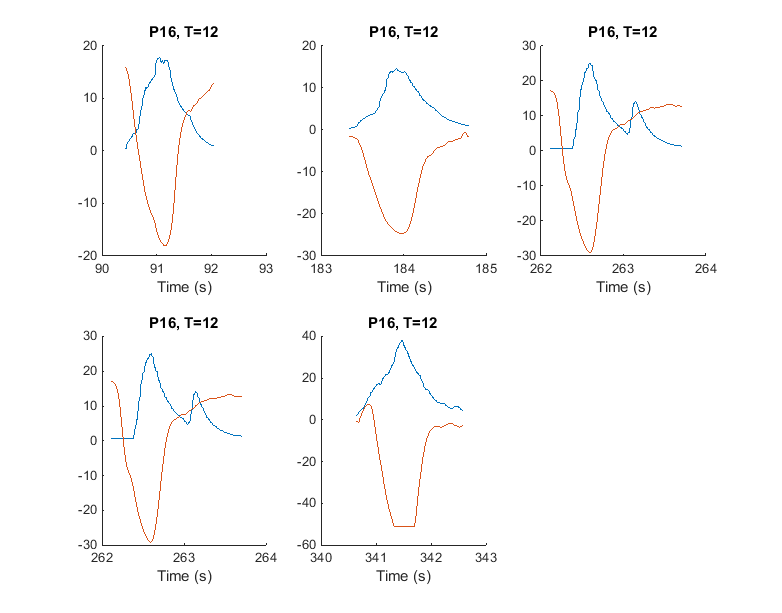


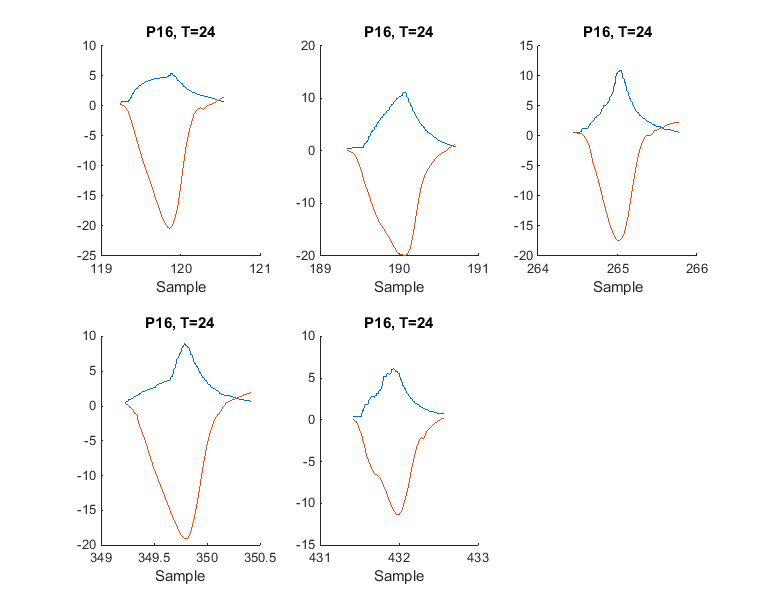


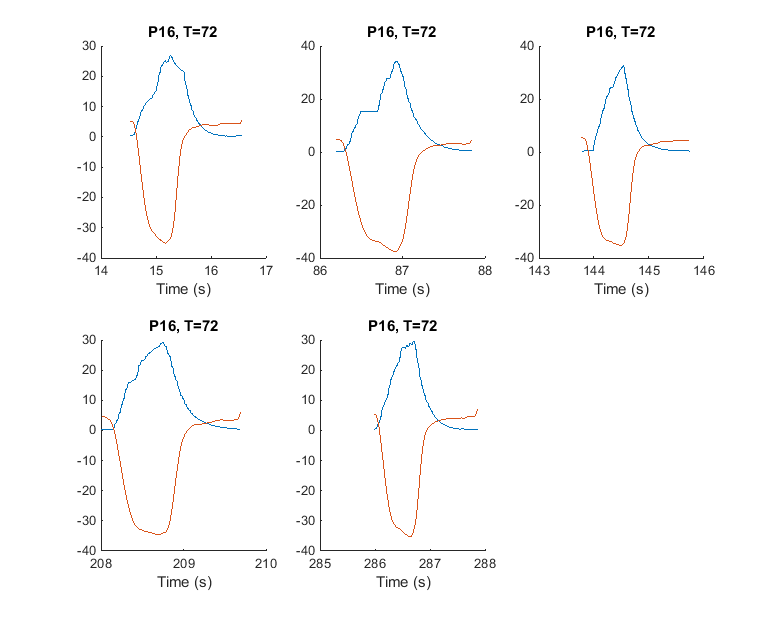


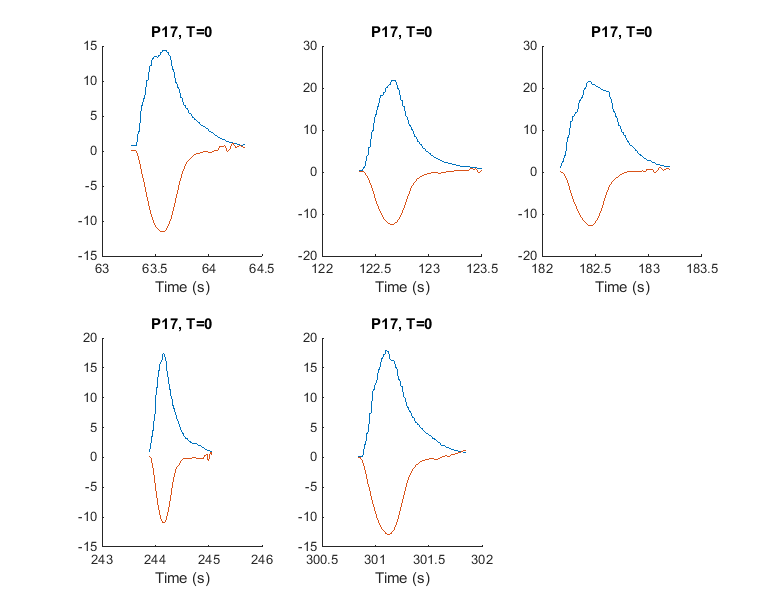

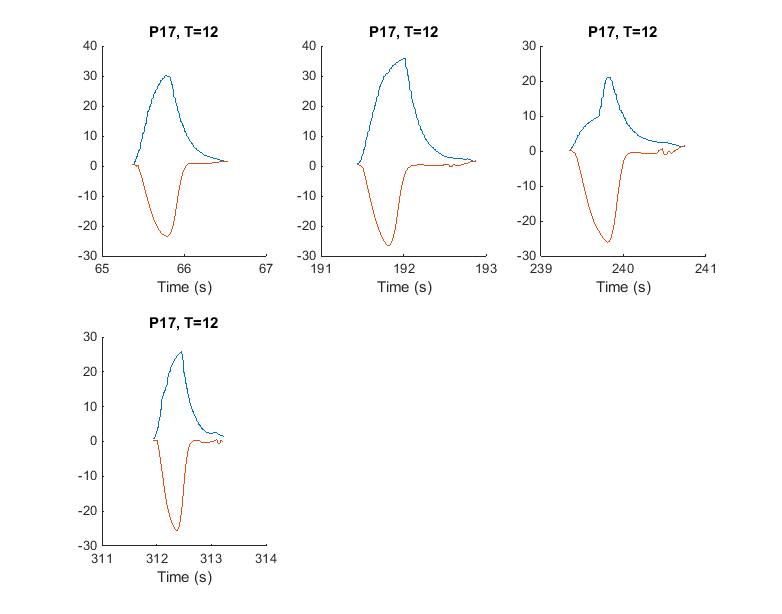


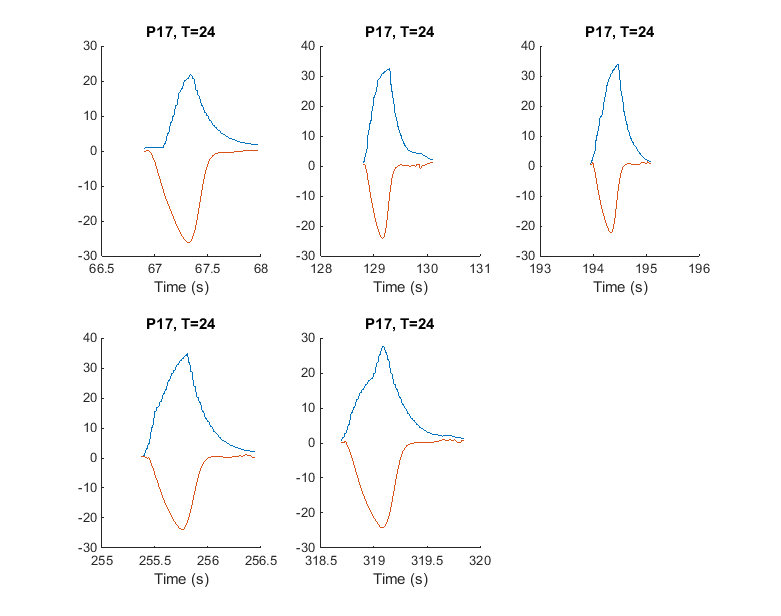

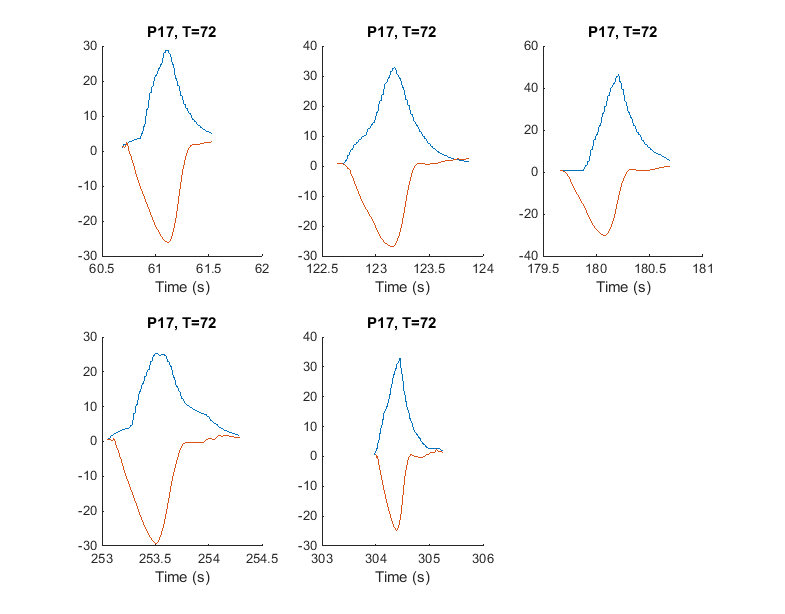


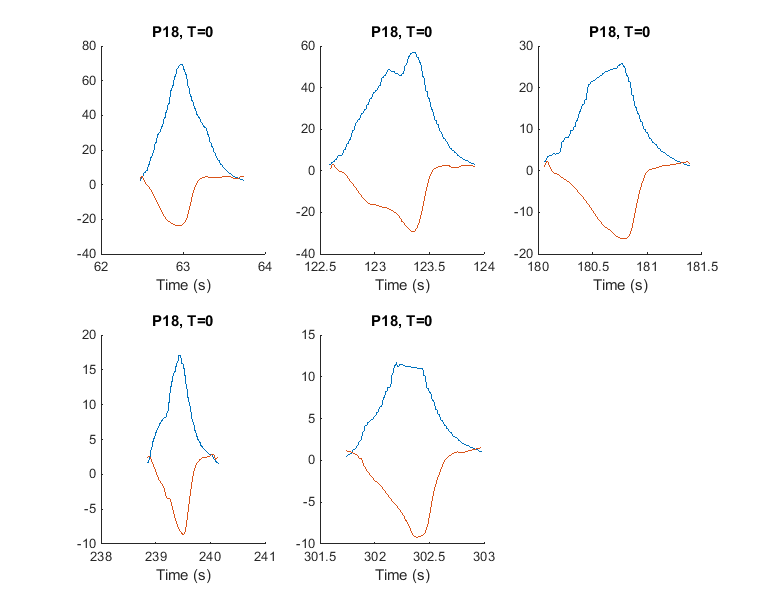


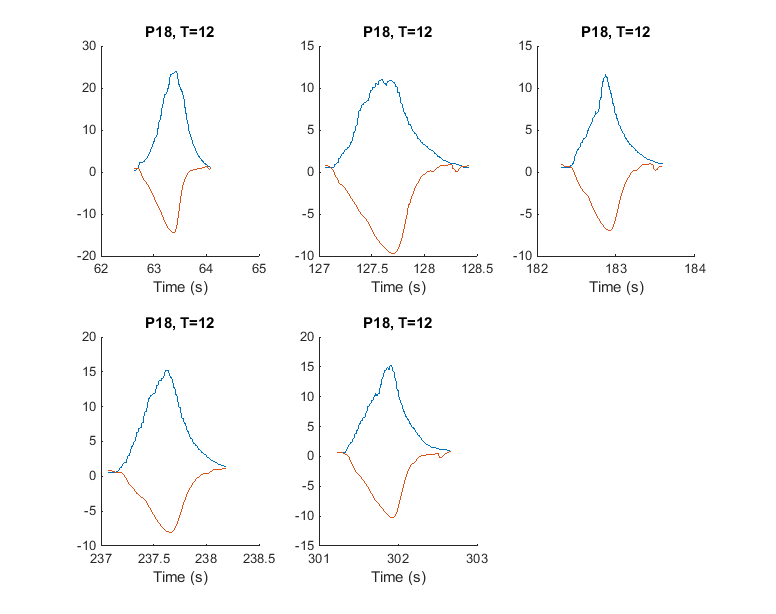


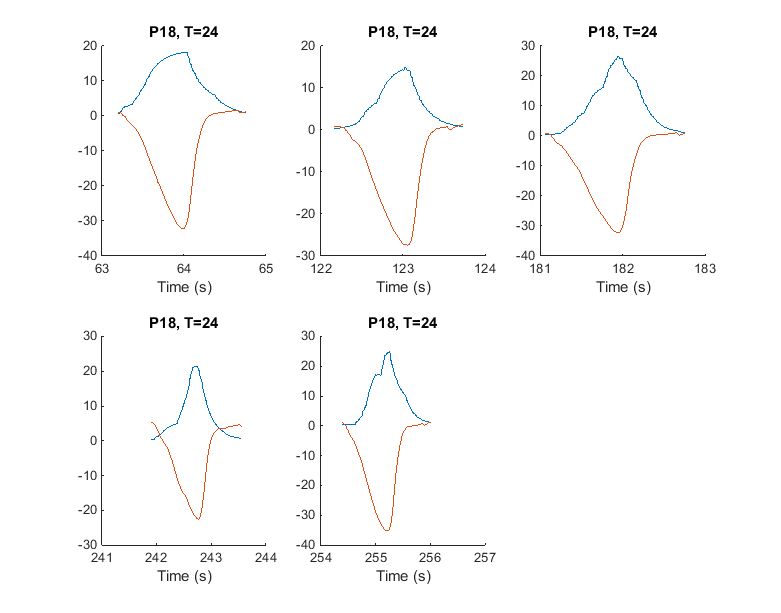


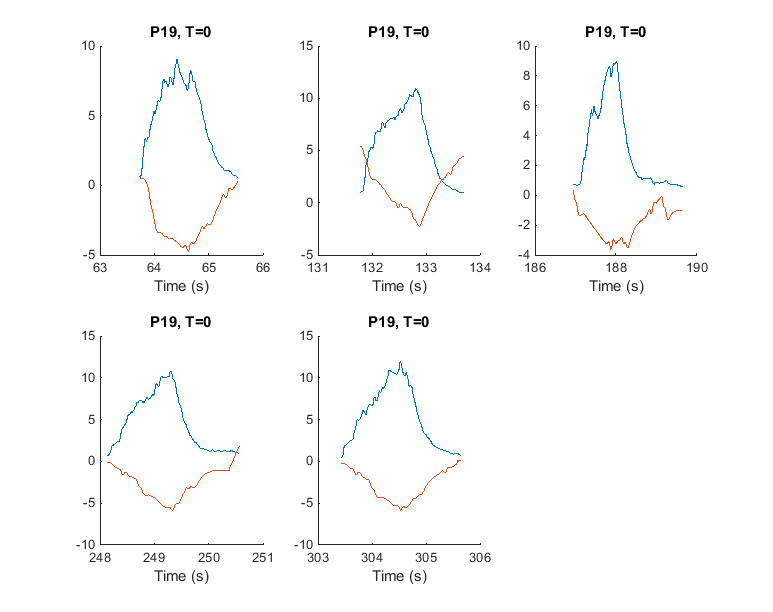


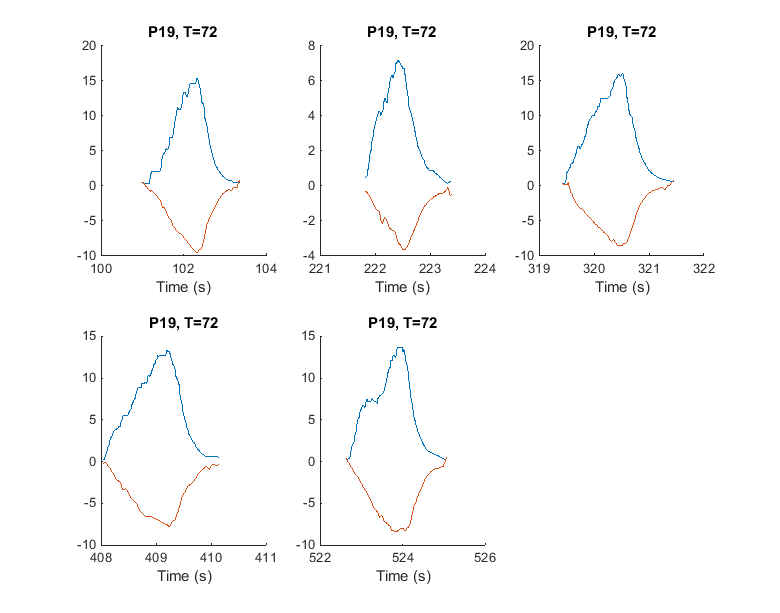


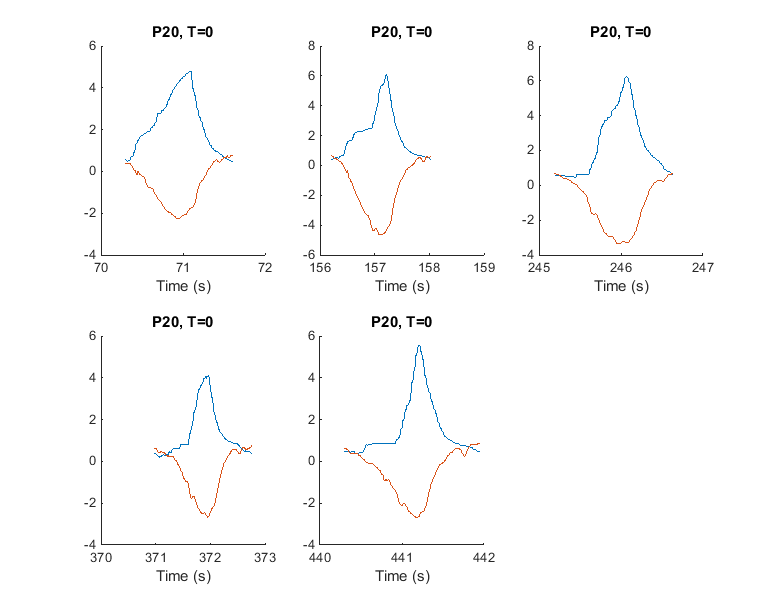


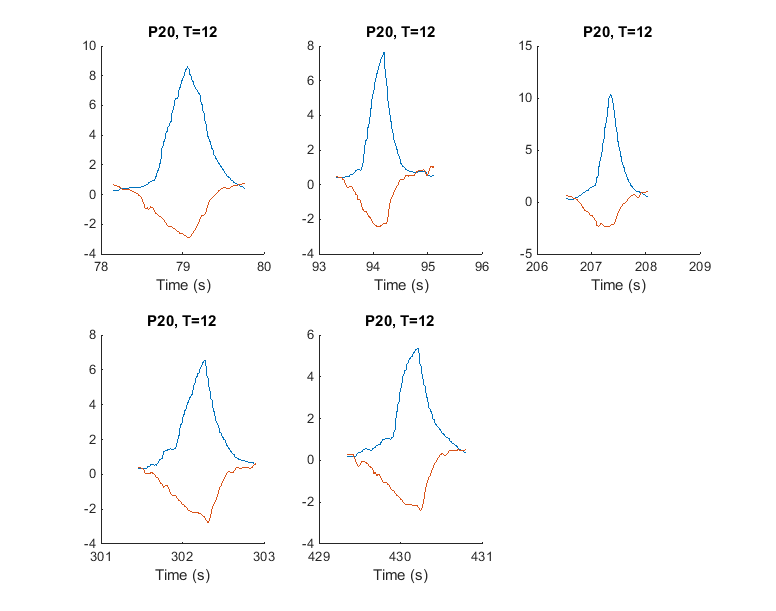


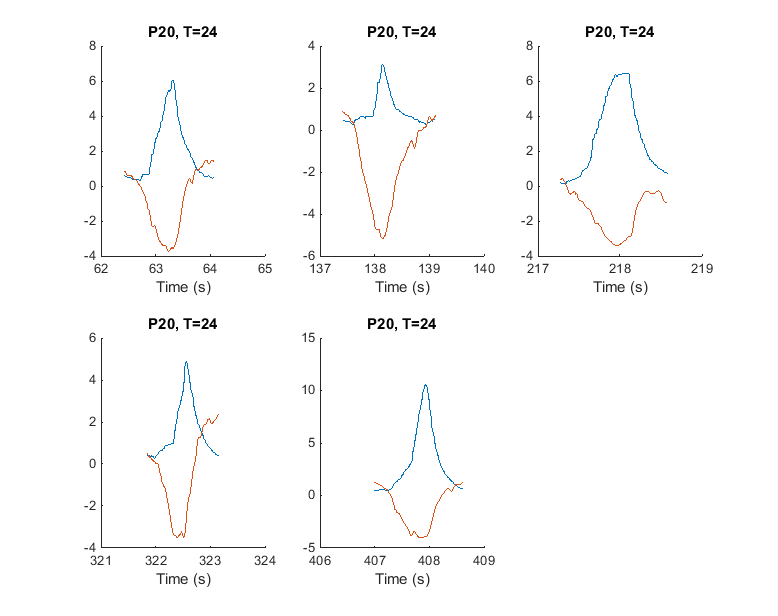


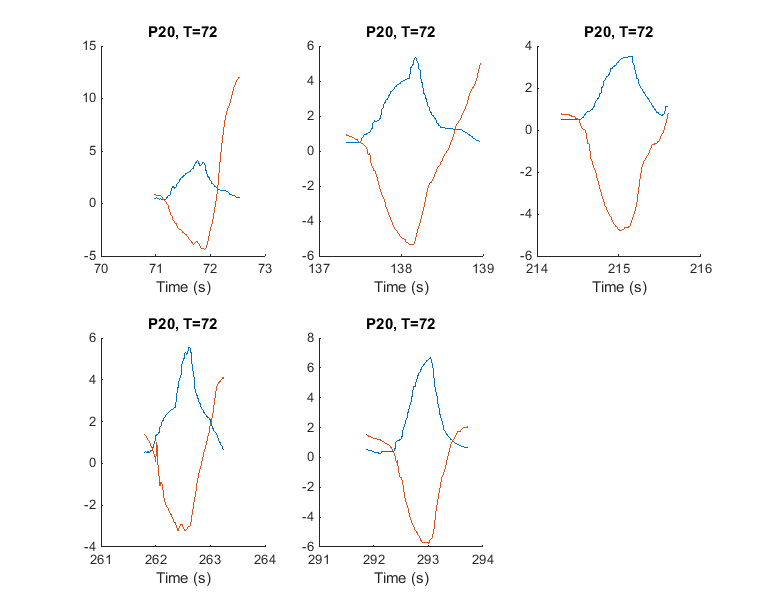


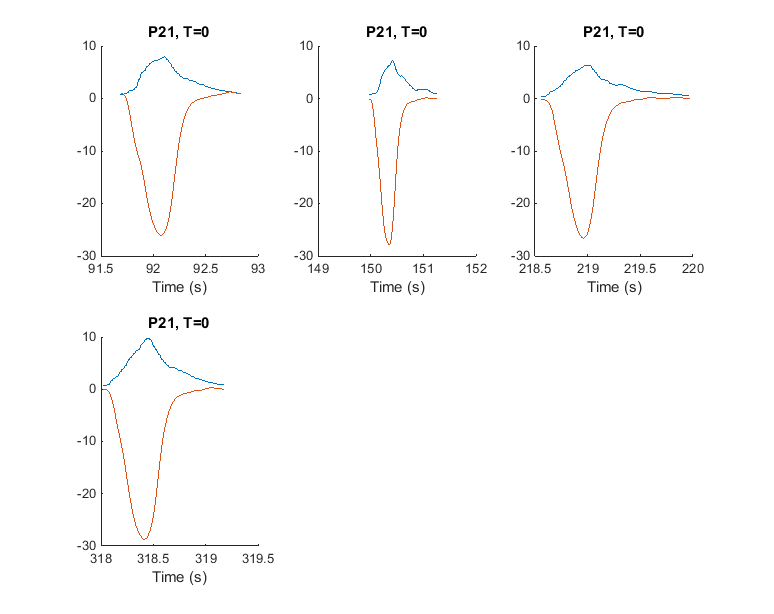


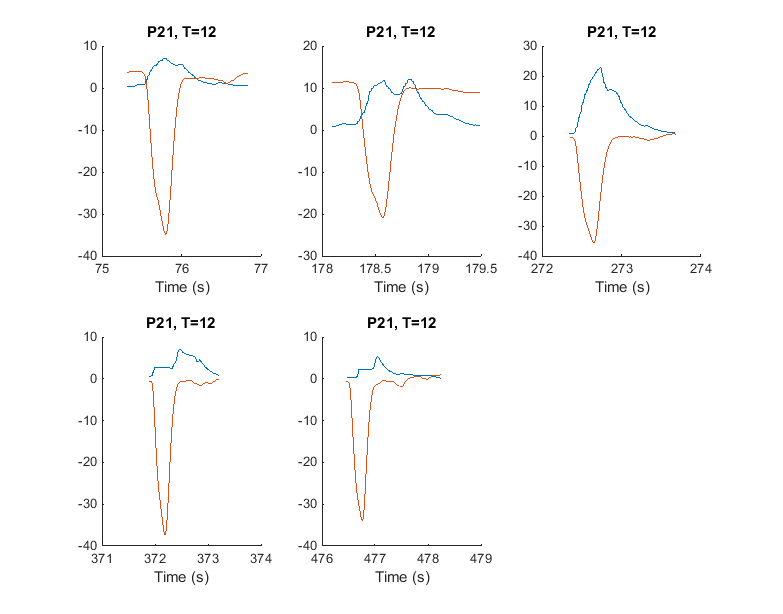


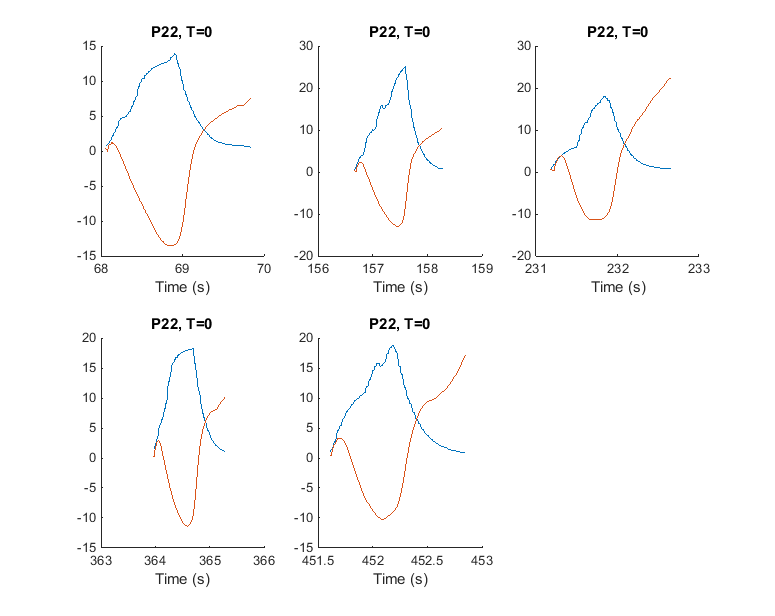


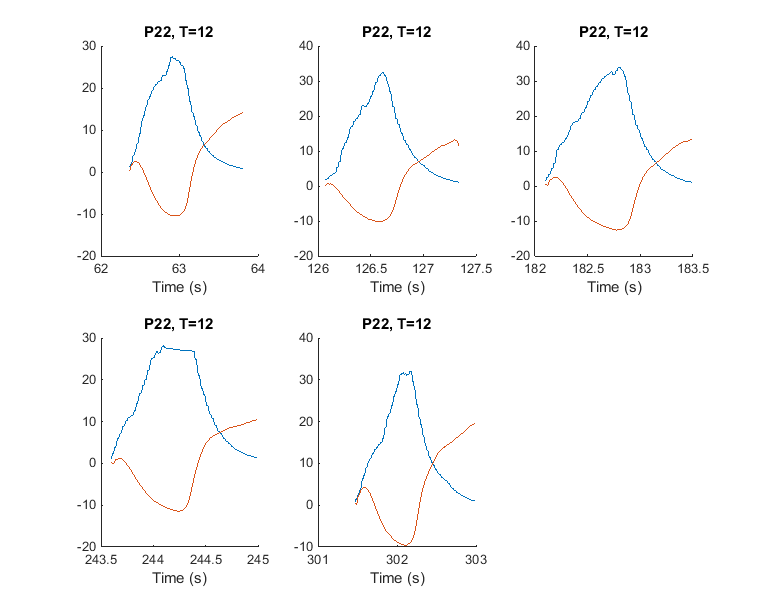


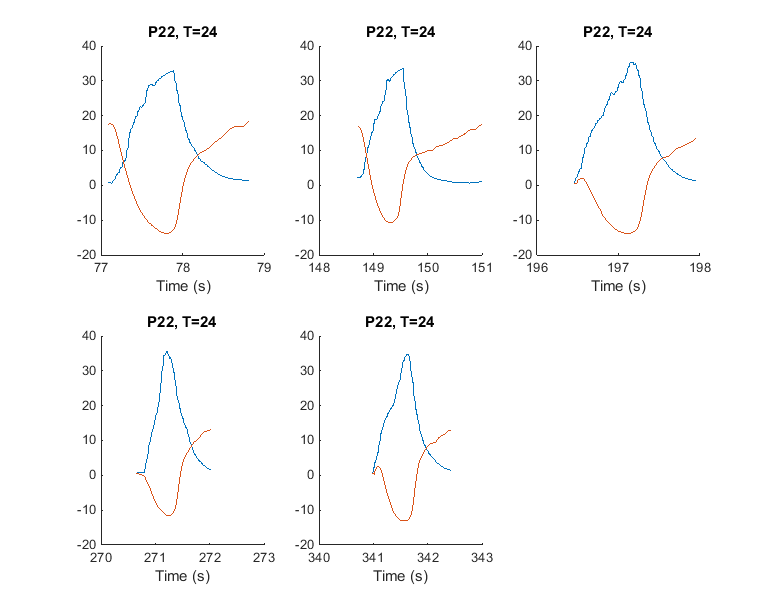


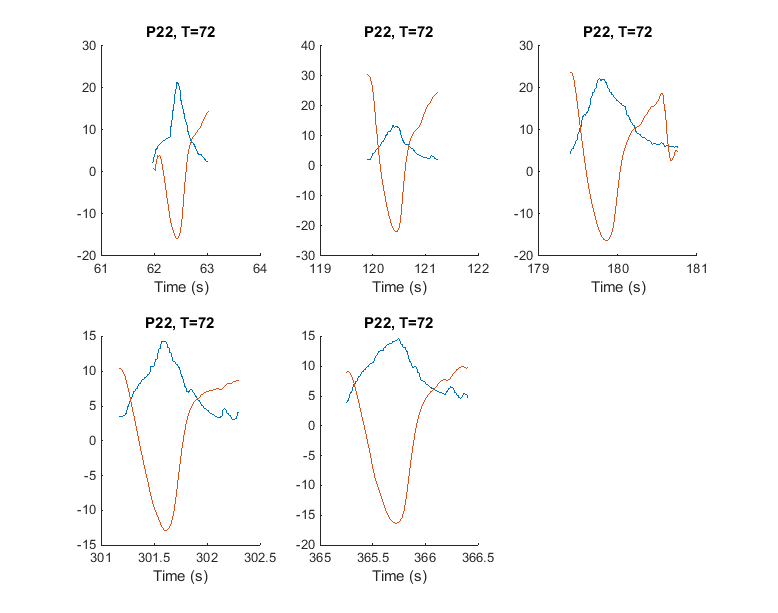


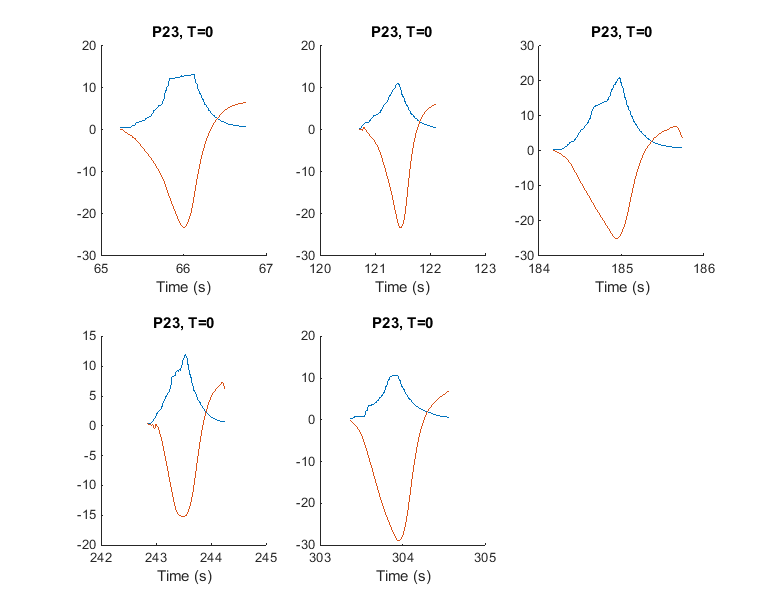


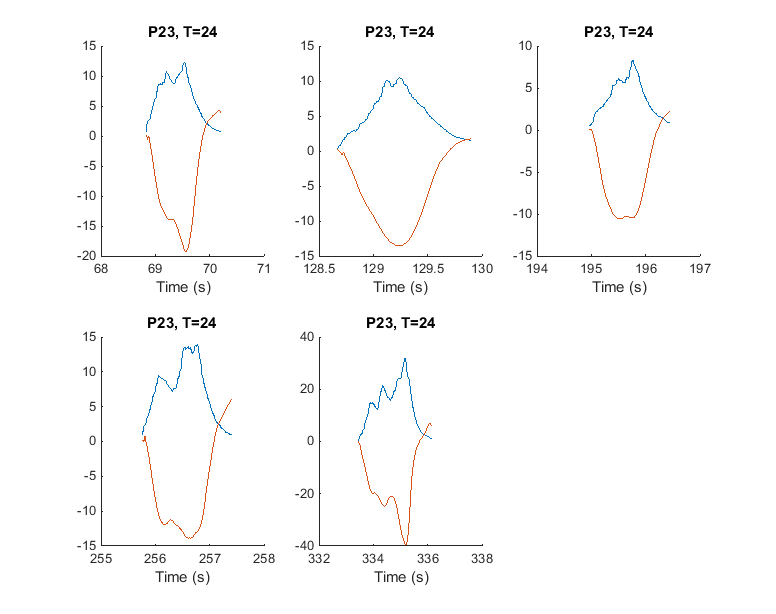


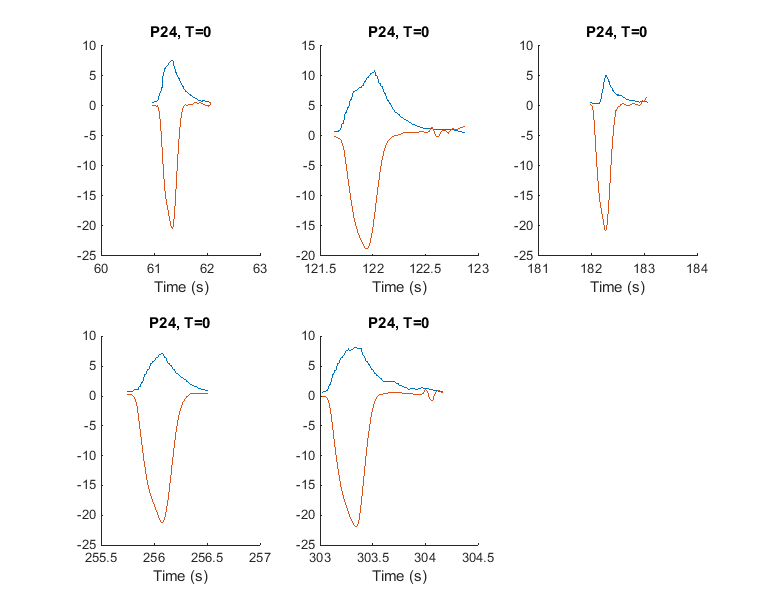


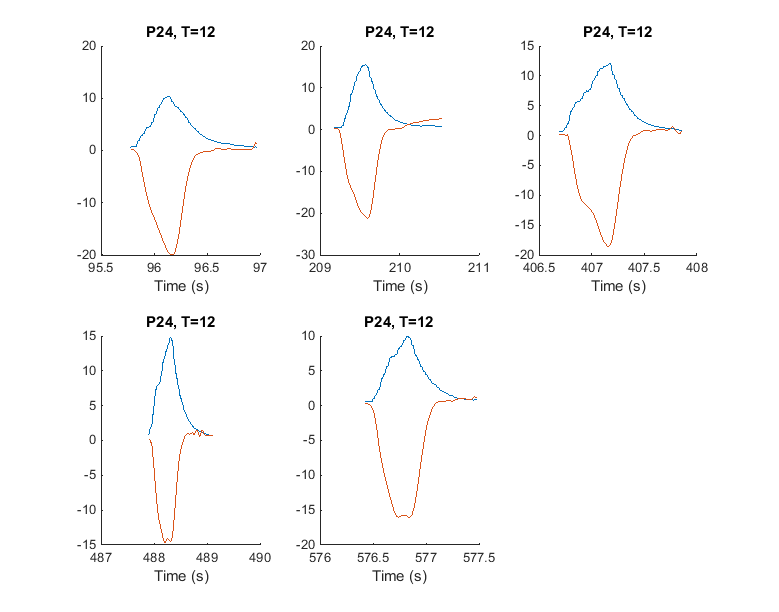


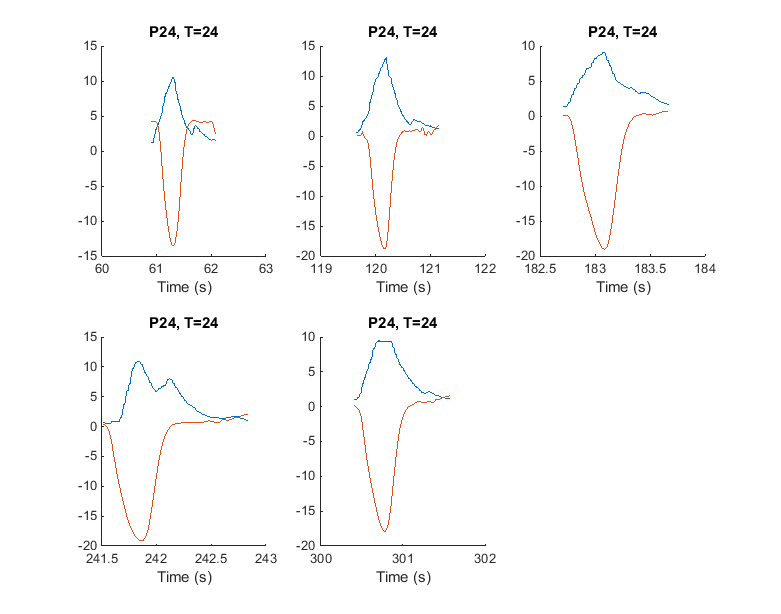


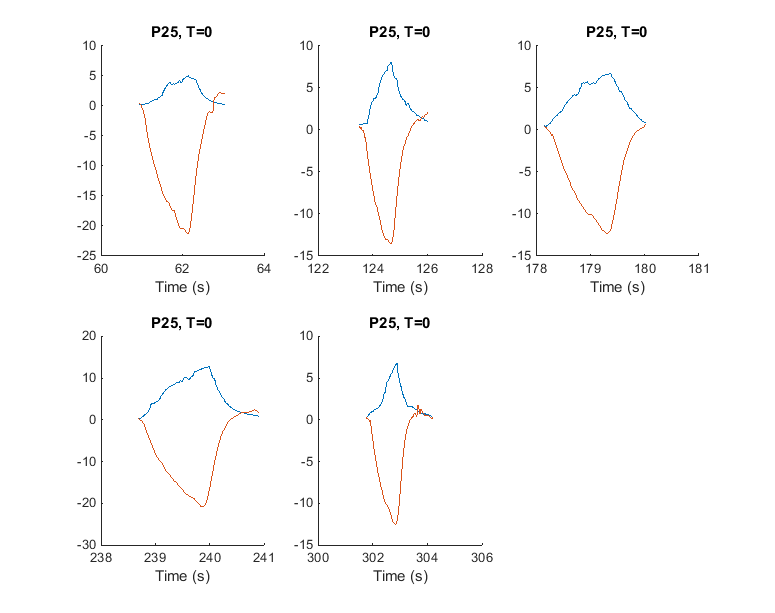

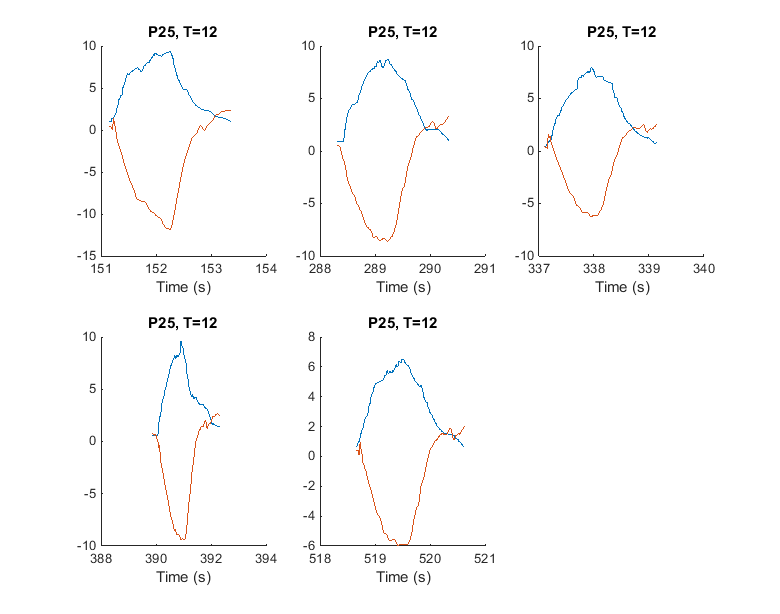


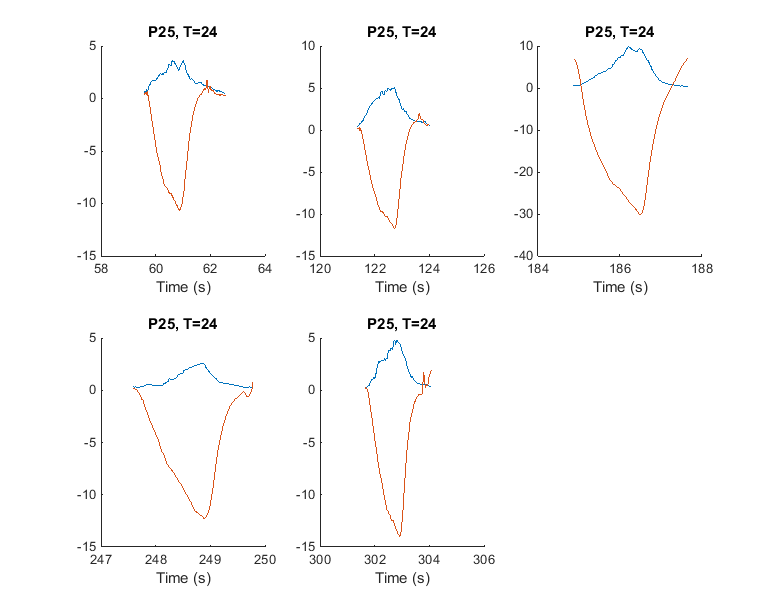


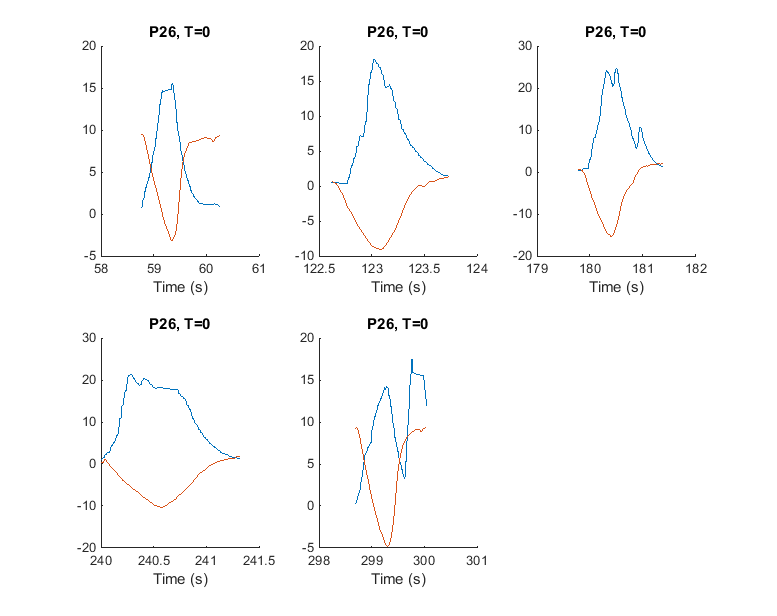

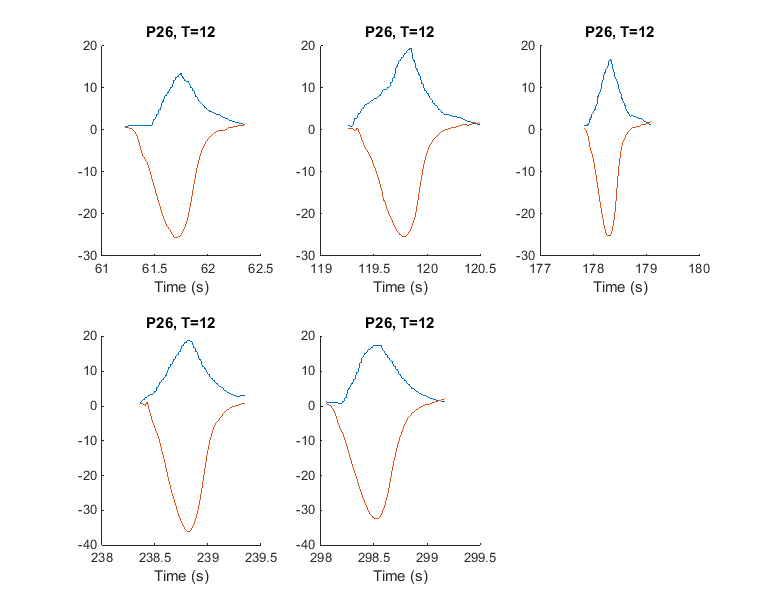


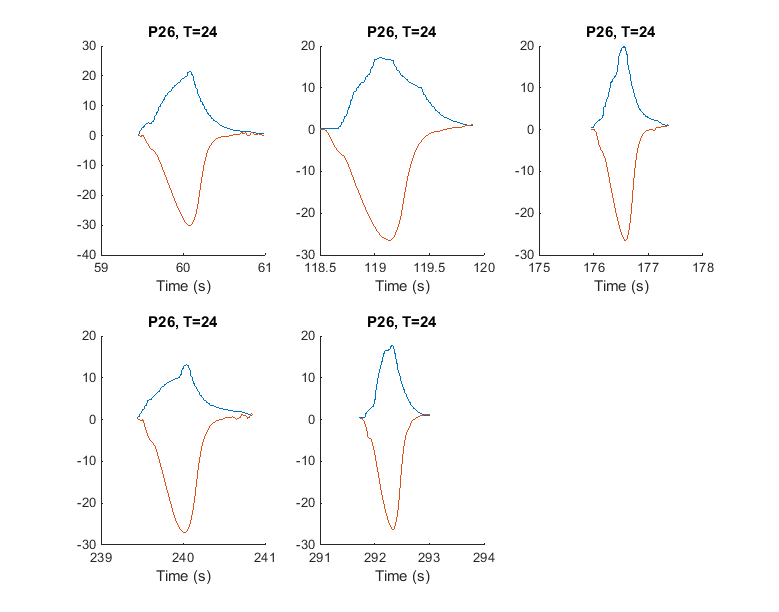


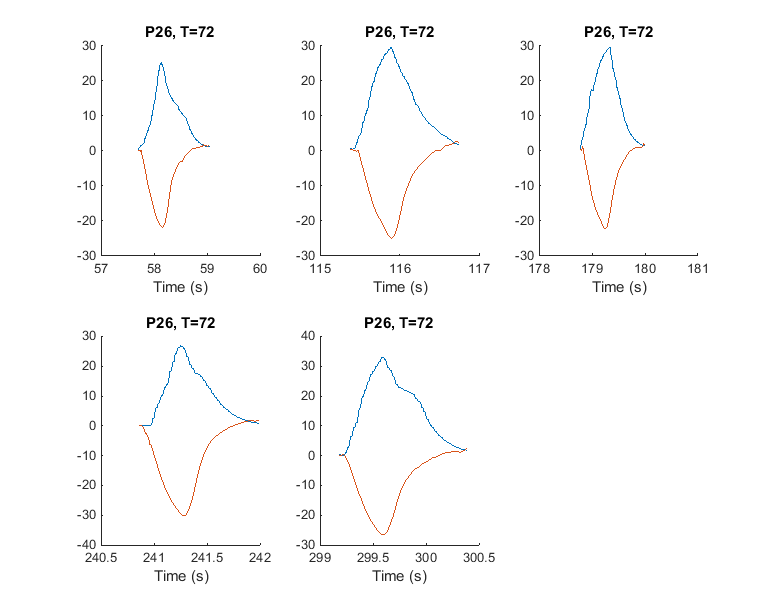


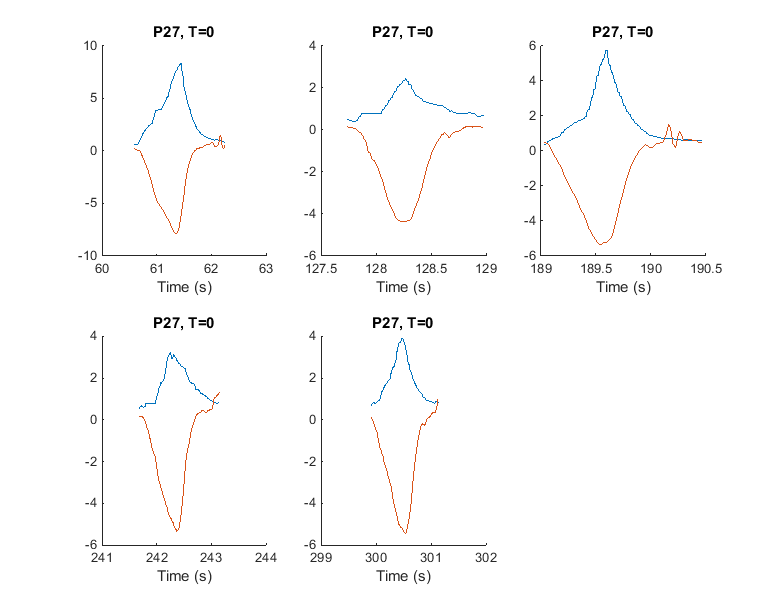


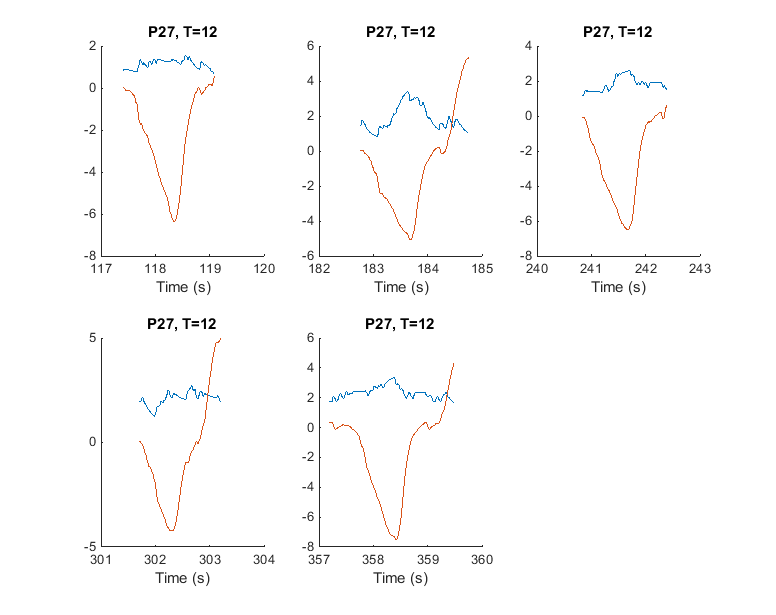


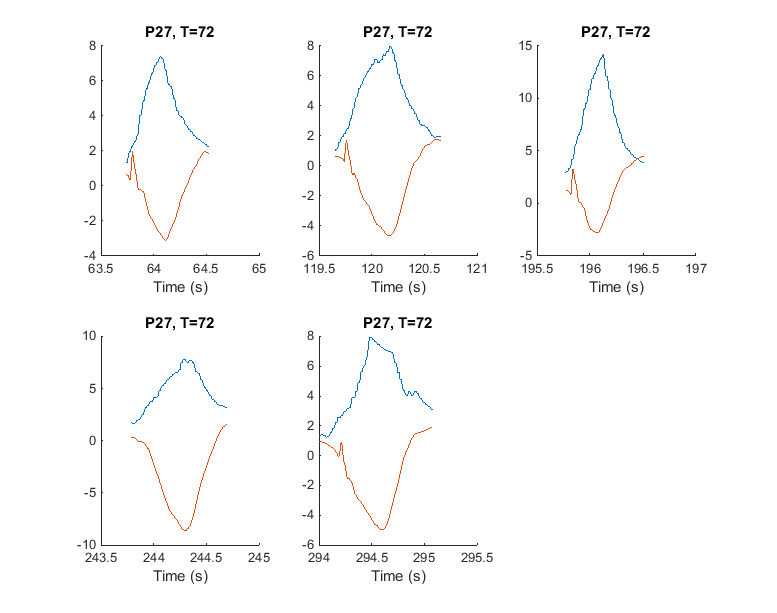


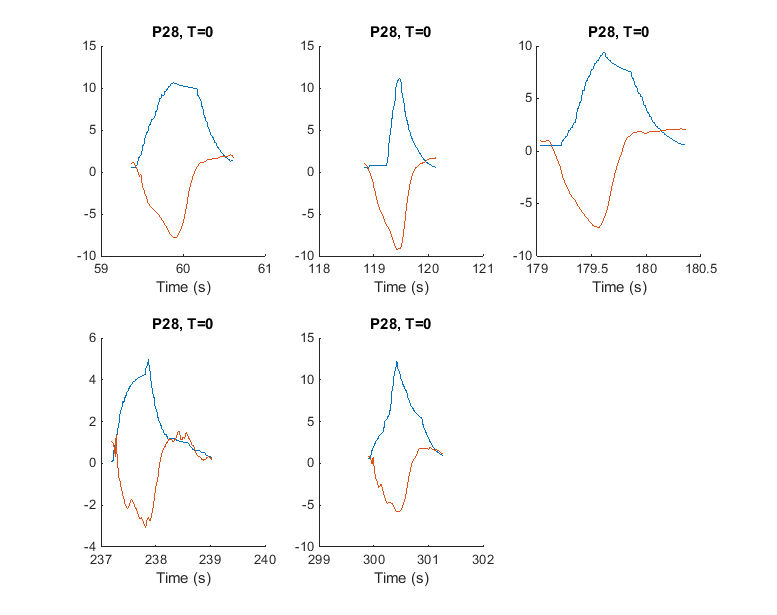


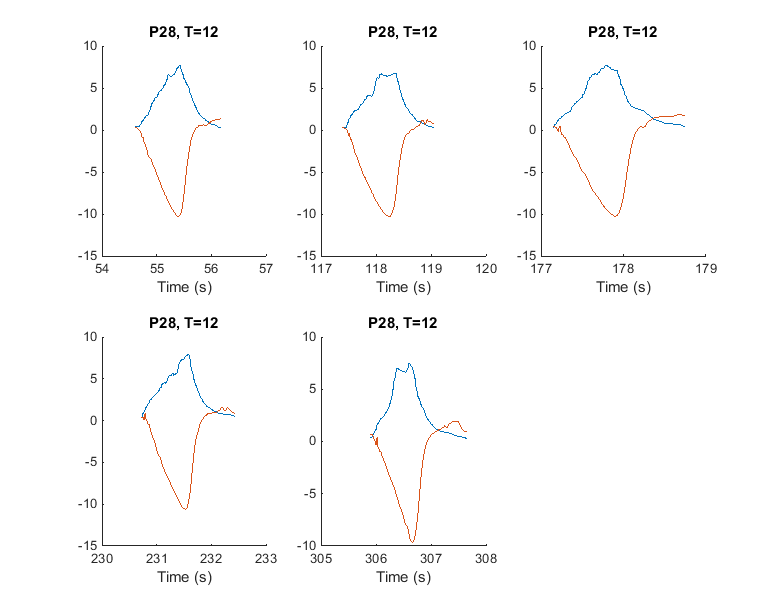


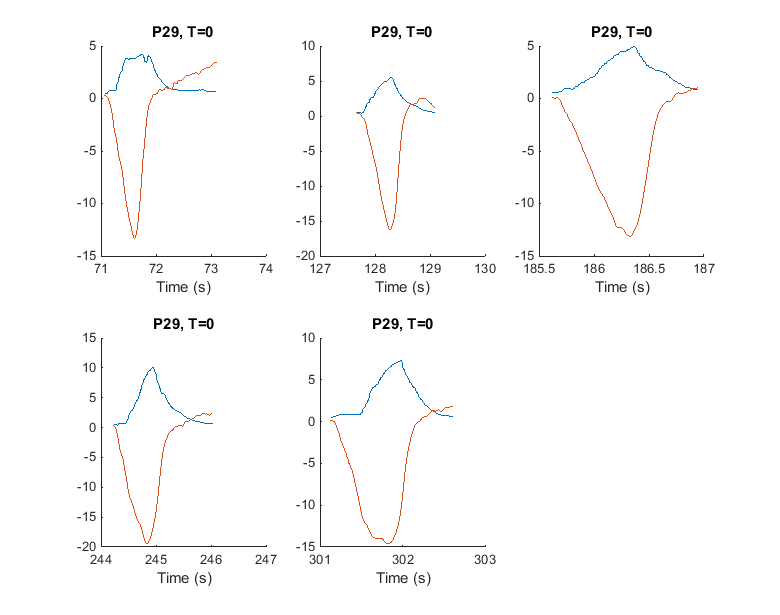


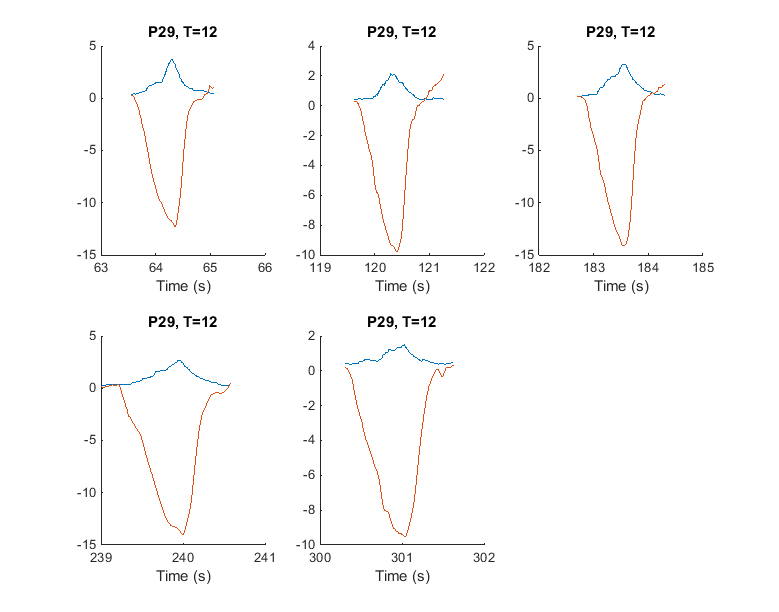


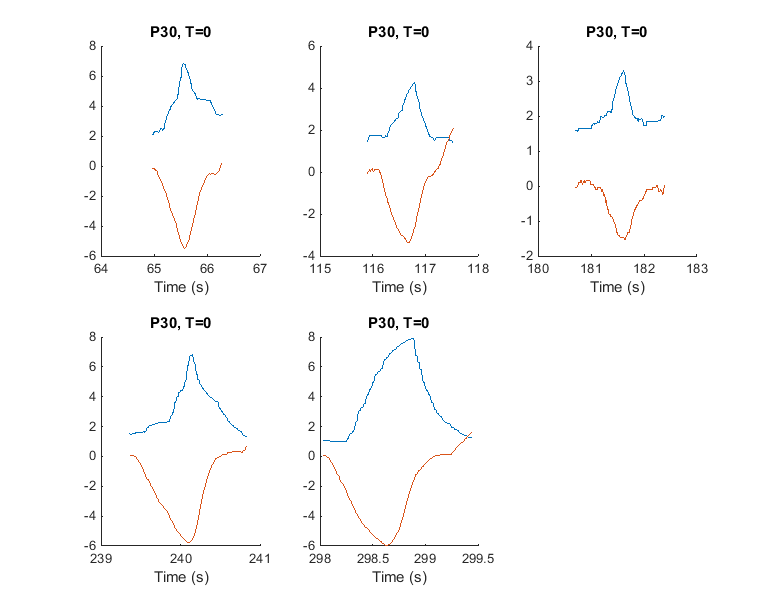


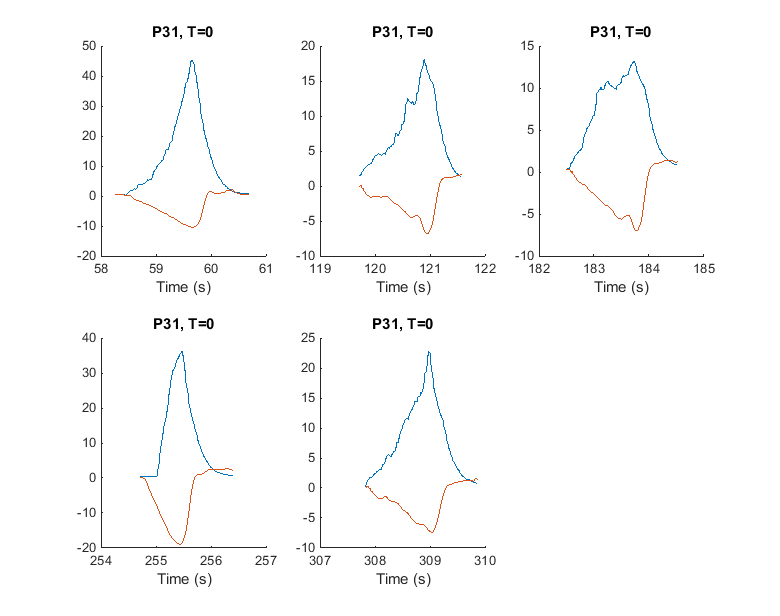


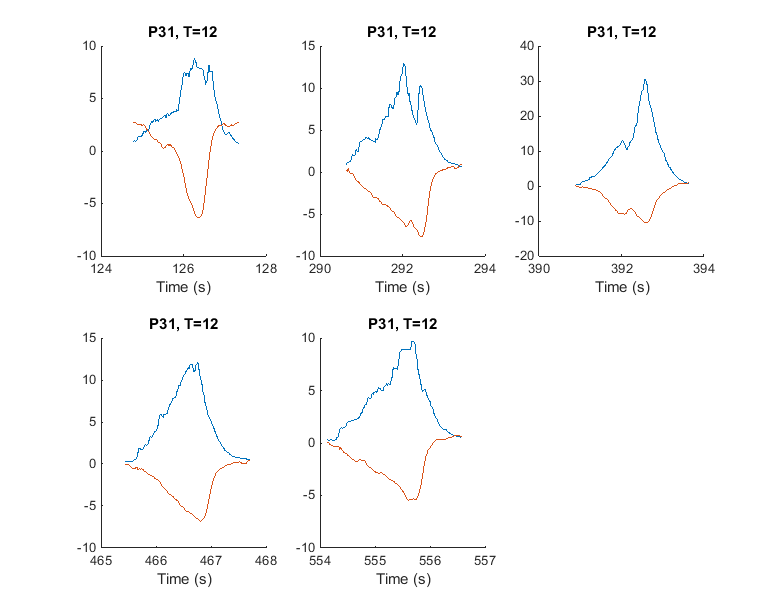


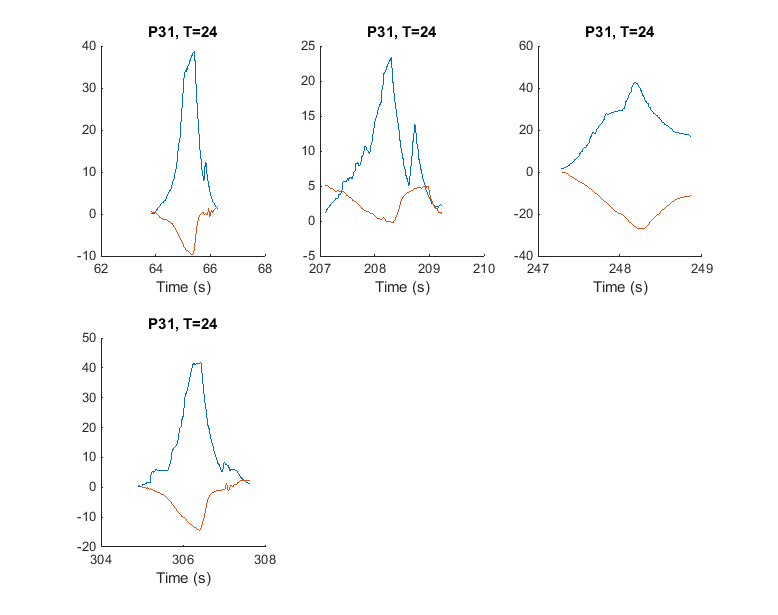


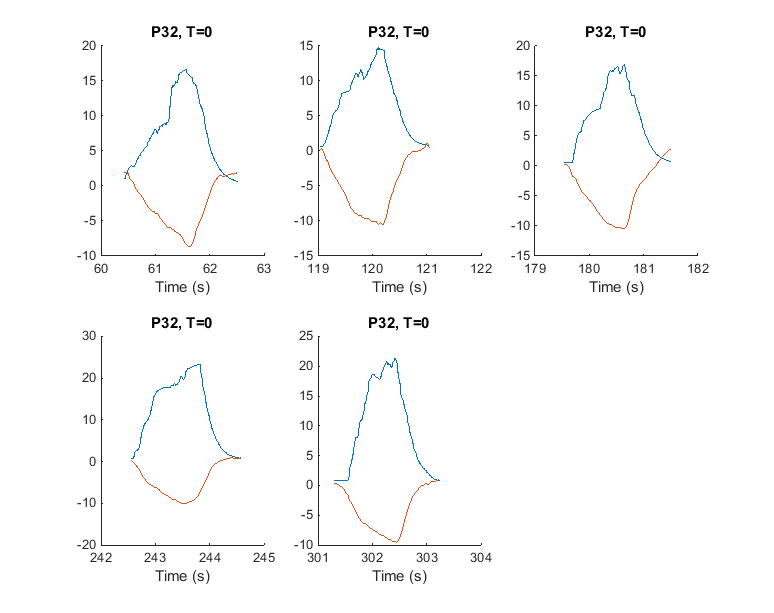


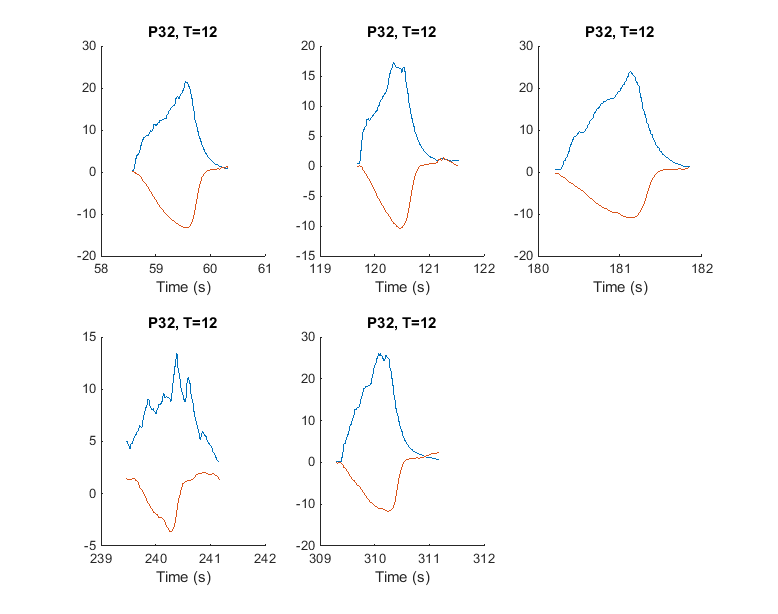


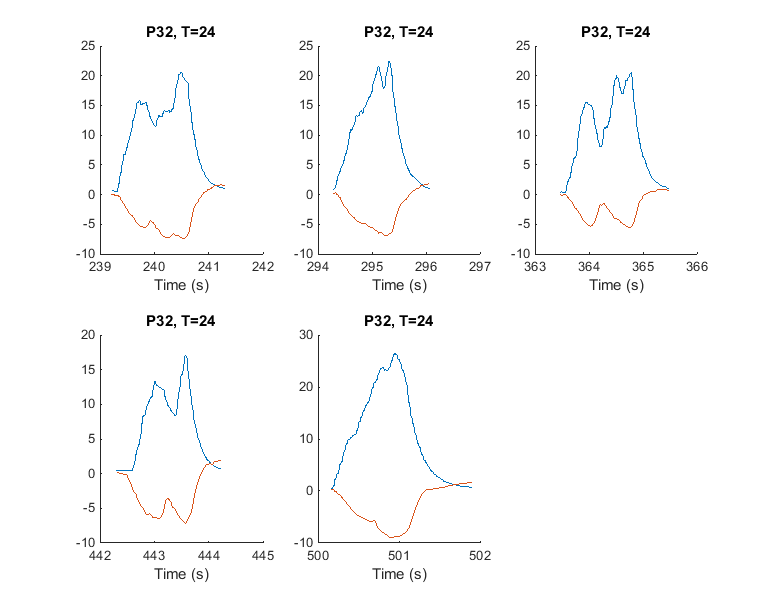


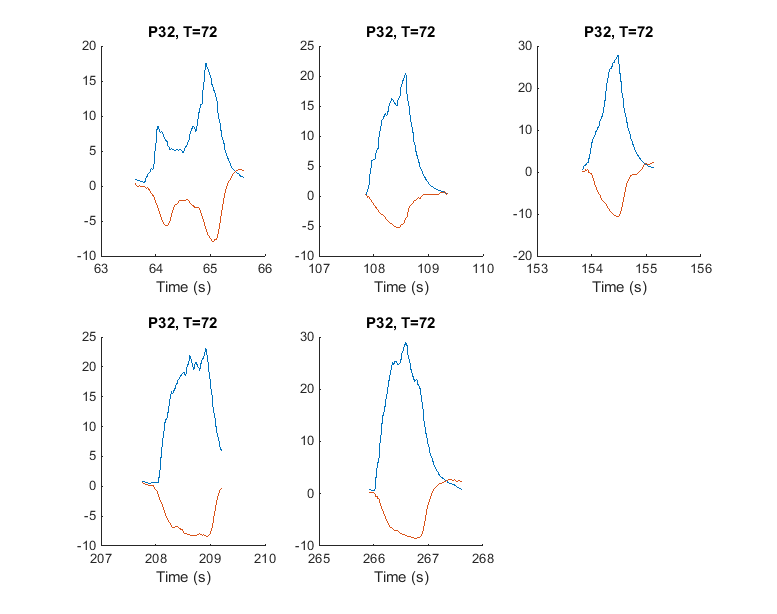


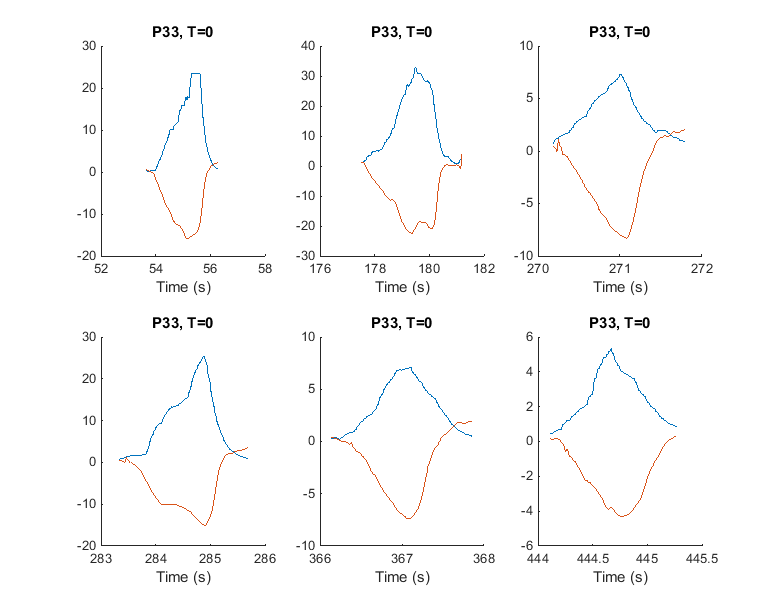


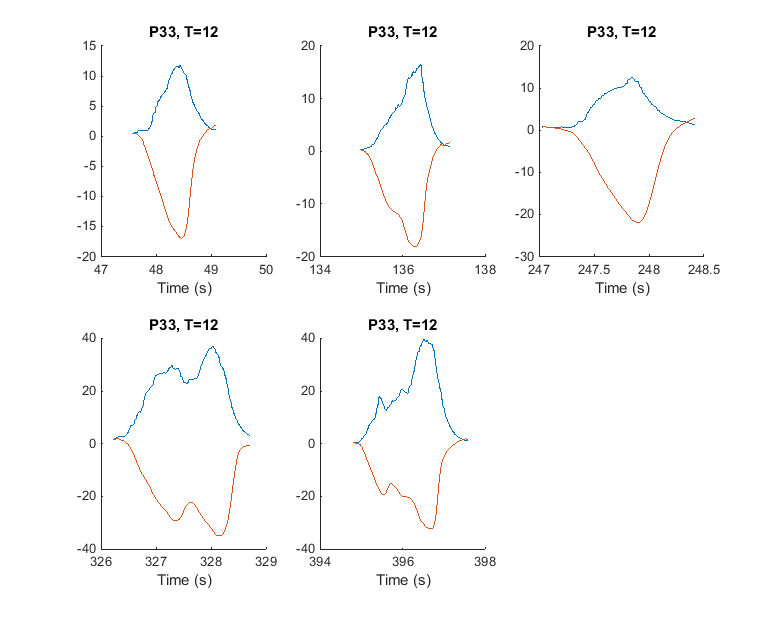


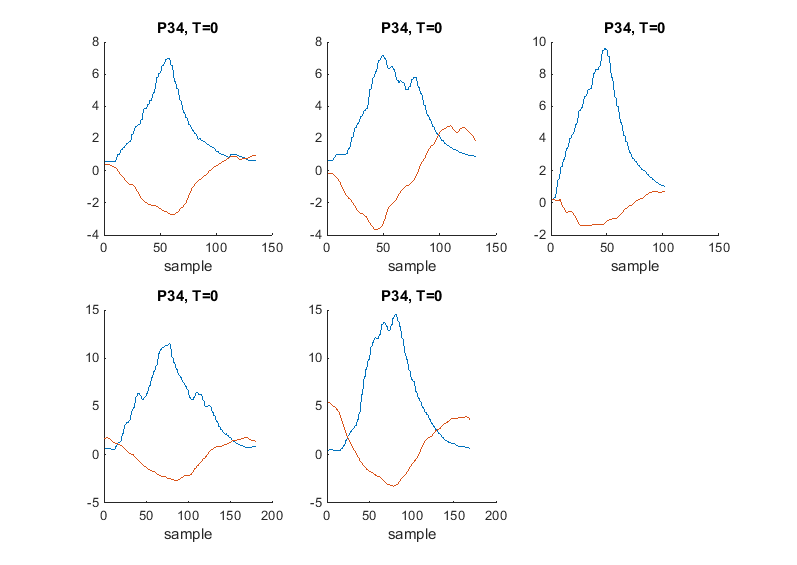


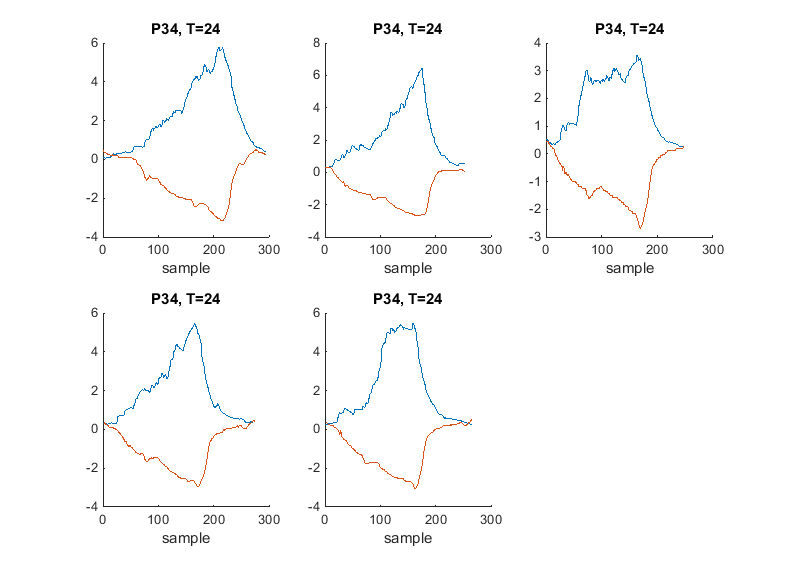


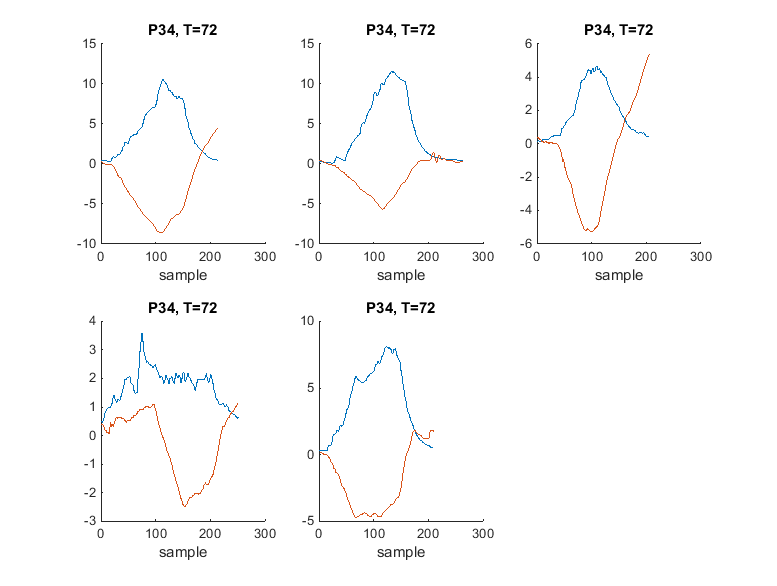


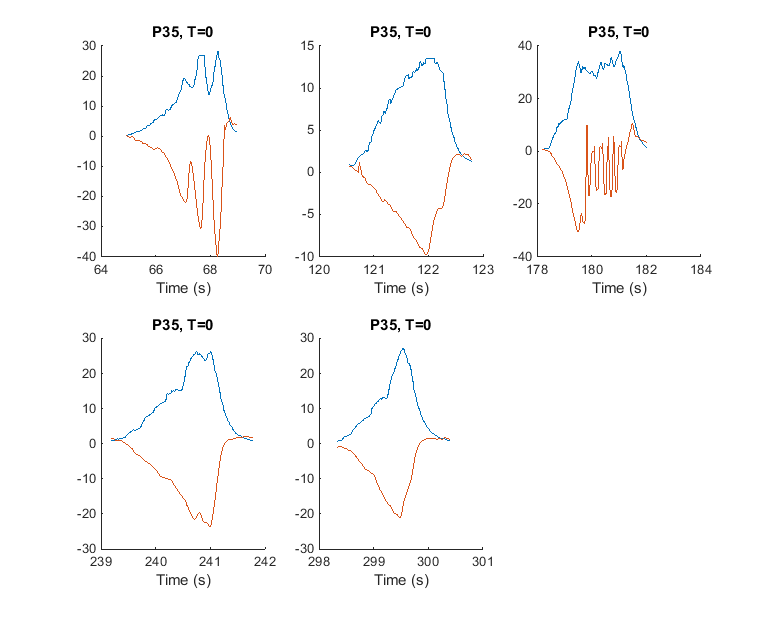

Supplement: Supplementary file 2 — Overview of the five repeated occlusions in each individual patient. The blue line represents the EAdi signal expressed in microvolts. The orange line represents the airway pressure (Paw) expressed in cmH2O. (DOCX 1942 kb) [file 13054_2018_2172_MOESM2_ESM.docx]
