# Supplementary material for: Estimation of the diaphragm neuromuscular efficiency index in mechanically ventilated critically ill patients
Source: Crit Care. 2018 Sep 27;22:238. doi: 10.1186/s13054-018-2172-0 (PMC6161422; doi:10.1186/s13054-018-2172-0)
Supplement: Supplementary file 3 — Extensive waveform analyses. This file gives an overview of the extensive waveform analyses that are performed, in order to objectively detect and exclude non-physiological EAdi waveforms. (DOCX 36 kb) [file 13054_2018_2172_MOESM3_ESM.docx]

**Additional File 3.**

The repeatability coefficient NMEoccl of 82.6% was deemed insufficient for clinical use. Upon visual inspection it appeared that some of the EAdi waveforms exhibited a rather non-physiological shape: a plateau during inspiration (while an increase would be expected) or at maximum inspiration, or a delay in increase of EAdi compared to the decrease in Paw (**see Figure 3 main manuscript**). It was assumed that this might be the result of suboptimal ventilator software for EAdi processing and filtering, based on electrocardiogram (ECG) replacements, which might contribute to the high variability of NMEoccl.

**Figure 3 main manuscript** shows the most common non-physiological EAdi waveforms observed during the end-expiratory occlusions. Often, a certain change in the slope was observed at random time points during the inspiratory phase (**Figure 3 main manuscript – A and D)**. In addition, it was demonstrated that the EAdi and Paw signals did not always start at the same time point. Most of the time this was due to an apparent ‘delay’ in the EAdi signal (**Figure 3 main manuscript – B)**, which differed in duration and timing between five repeated measurements within a patient (see for example P4 T=12 in **Supplementary, Additional File 2**). Therefore, the delay could not be the result of a technical problem in the data acquisition.

It was reasoned that the non-physiological EAdi waveforms are the result of inappropriate processing of EAdi recordings within the ventilator software. Besides measuring the diaphragmatic electrical activity, the sensors imbedded in the EAdi catheter measure other electrical activity nearby as well, such as cardiac electrical activity and esophageal movement. Since the cardiac electrical activity is much stronger than the diaphragm electrical activity, additional measures must be taken to subtract the ECG signal (mainly QRS complex) from the EAdi signal to ensure accurate diaphragm activity recordings. Computer algorithms within the ventilator software (patented by Maquet, no details available) are responsible for this filtering process. Inappropriate filtering could lead to a certain loss of data. Since we did not have access to the raw EAdi data, it was impossible to make assumptions about the underlying raw data, i.e. true course or peak of the EAdi signal. Calculating NMEoccl based on these inappropriate filtered EAdi waveforms will lead to incorrect NMEoccl values and thereby to a variability that is disproportionally high. Therefore, we aimed to develop criteria to mathematically detect and exclude these non-physiological EAdi waveforms and thereby improve NMEoccl variability.

**Mathematical approaches to detect and exclude non-physiological EAdi waveforms**

It was hypothesized that all zero or negative EAdi slopes with a certain sample length anytime during the inspiratory phase are ‘non-physiological’ and should therefore be excluded for further analysis. To detect these zero or negative EAdi slopes, a continuous slope of the inspiratory increase in EAdi was calculated first, as the derivative of the EAdi. Then, it was tried to objectively detect the zero or negative EAdi slope with a predefined sample length. However, due to a wide variation in the duration of the irregularities between maneuvers and patients, it was not possible to objectively define this ‘sample length’ and thereby exclude maneuvers based on these criteria. In addition, sometimes a sudden change in slope of the inspiratory EAdi waveform was seen (not accompanied by a change in Paw) while the slope remained positive **(Figure 1a below)**. These factors together ensure that it was impossible to define or standardize ‘change in slope’.


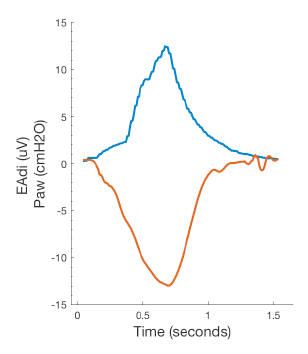


**Figure 1a**. Example of a subtle change in slope of the EAdi waveform.

Apparently, it was possible to objectively identify rough irregularities such as deep inflections of the EAdi waveform during the inspiratory phase or EAdi peaks that were horizontal for a certain time period during the maneuver **(Figure 3 main manuscript – C).** However, removing these waveforms in the analyses did not improve the variability in NMEoccl sufficiently, as numerous artifacts could not be objectively detected using mathematical approaches.

Simply remove all waveforms with any kind of subtle irregularities will lead to an unacceptable low amount of remaining waveforms and to observer bias.

From a physiological perspective, EAdi should increase during an inspiratory effort and Paw should decrease. Therefore it is reasonable to remove only parts of the EAdi and corresponding Paw waveforms that did not meet this theory, instead of excluding the whole maneuver from the analyses. Parts of the waveform that did not meet the following criteria were removed: 1) difference between the current and previous sample of EAdi (EAdi_x_ – Eadi_x-1_) > 0 and 2) similarly Paw_x_ – Paw_x-1_ < 0. It was hypothesized that with this method ECG replacements could be eliminated and thereby reducing the variability between occlusion maneuvers. After removing the parts of both waveforms that did not meet the criteria, the remaining parts of both waveforms were plotted against each other and its slope was considered NMEoccl. The slope was estimated using a linear regression analysis according to the following equation, in which *a* is the slope of the linear regression which can be considered the NME: Paw = a * EAdi + b.

This method represents a time-independent relation of EAdi and Paw, in which only artifacts were eliminated. However, this method did not improve RC NMEoccl: 90.9%. After including only those slopes with a determination coefficient > 80%, RC NMEoccl repeatability improved moderately to 63.4%.

In conclusion, different mathematically approaches were invented and tested, but did not reduce NMEoccl variability to acceptable values. Therefore we pragmatically continued with the method described in the main manuscript.
