# Supplementary material for: Estimation of the diaphragm neuromuscular efficiency index in mechanically ventilated critically ill patients
Source: Crit Care. 2018 Sep 27;22:238. doi: 10.1186/s13054-018-2172-0 (PMC6161422; doi:10.1186/s13054-018-2172-0)
Supplement: Supplementary file 4 — Correlation of clinical parameters and NMEoccl variability at T = 0. First, the coefficient of variation (CoV) was calculated for each patient. The median CoV for NMEoccl at T = 0 was 23.1% (IQR 18.7–29.9%). The study population was divided in two groups, with the CoV higher or lower than the median, respectively. CoV = coefficient of variation; COPD = chronic obstructive pulmonary disease; EAdi = electrical activity of the diaphragm; HR = heart rate; IQR = interquartile range; NAVA = neutrally adjusted ventilatory assist; RASS = Richmond agitation sedation scale; RR = respiratory rate; VT = tidal volume. (DOCX 18 kb) [file 13054_2018_2172_MOESM4_ESM.docx]

| **Additional File 4. Correlation of clinical parameters and NMEoccl variability at T=0.** | | | | |
| --- | --- | --- | --- | --- |
| **Clinical parameters** | **All (N=31)** | **CoV above median (N=15)** | **CoV below median (N=16)** | **P-value** |
| RASS, median [IQR] | -1 [0/-2] | -1 [0/-1.5] | -2 [-0.5/-3] | 0.086 |
| Sedatives, n= (%) | 8 (25.8%) | 4 (26.7%) | 4 (25%) | 0.919 |
| Opioids, n= (%) | 13 (41.9%) | 6 (40%) | 7 (43.8%) | 0.839 |
| Use of steroids, n= (%) | 8 (26%) | 5 (33%) | 3 (19%) | 0.374 |
| RR (per minute), median [IQR] | 25 [17-30] | 20 [16-28] | 26 [19-31] | 0.151 |
| VT (ml), median [IQR] | 450 [395-438] | 450 [415-490] | 420 [389-545] | 0.521 |
| NAVA level, median [IQR] | 0.8 [0.5-1.3] | 0.8 [0.6-1.4] | 0.7 [0.2-1] | 0.056 |
| EAdi (µV), median [IQR] | 13.0 [7.5- 20.7] | 12.2 [7.3-19.1] | 13.0 [9.0-19.1] | 0.546 |
| Low Eadi (<5µV), n= (%) | 2 (6.5%) | 2 (13.3%) | 0 (0%) | 0.164 |
| Duration of MV on T=0 (days), median [IQR] | 10 [8.5-18.5] | 9 [4.5-19] | 11.5 [9-17] | 0.292 |
| HR (beats/min), median [IQR] | 84 [75-95] | 80 [72-88] | 88 [79-95] | 0.258 |
| Atrial fibrillation, n= (%) | 1 (3,2%) | 0 (0%) | 1 (6,3%) | 0.333 |
| Temperature, median [IQR] | 37.7 [37-38.2] | 37.4 [36.9-37.8] | 37.8 [37.2-38.3] | 0.118 |
| COPD, n= (%) | 4 (12.9%) | 2 (13.3%) | 2 (12.5%) | 0.947 |
| Neuromuscular disease, n= (%) | 4 (12.9%) | 3 (20%) | 1 (6.3%) | 0.278 |
